# Direct observation of quantum scattering oscillations in the controlled Kr* + Rb cold collision

**DOI:** 10.1126/sciadv.aeh1789

**Published:** 2026-09-04

**Authors:** Yu-Chan Wang, Rui-Fan Wu, Hao Li, Zhao-Cong Shi, Guo-Min Yang, Ya-Fu Guan, Bin Zhao, Wei Jiang

**Affiliations:** ^1^ Hefei National Research Center for Physical Sciences at the Microscale and School of Physical Sciences, University of Science and Technology of China, Hefei 230026, China.; ^2^ Hefei National Laboratory, University of Science and Technology of China, Hefei 230088, China.; ^3^ Department of Chemistry, College of Science, Southern University of Science and Technology, Shenzhen 518055, China.; ^4^ Dalian Institute of Chemical Physics, Chinese Academy of Sciences, Dalian 116023, China.

## Abstract

Quantum interference in cold collisions has been predicted to dominate chemical dynamics at low temperatures. However, experimental observations have been largely confined to light-atom systems due to technical challenges. Here, we report direct observation of scattering oscillations in cold ionizing collisions between metastable krypton and rubidium atoms. By combining laser cooling with velocity-mapped ion imaging, we precisely controlled the collision energy from 15 millikelvin to 8 kelvin and detected the ionization products. Below 1 kelvin, pronounced quantum interference between partial waves emerges as the de Broglie wavelength approaches the interaction range. The angular oscillation period scales as $\theta_{p}\propto E^{-1/3}$, consistent with a Langevin capture model for van der Waals–dominated collisions. Moreover, interference patterns in the angular distribution–collision temperature spectrum are highly sensitive to the long-range potential shape and reveal subtle shape resonances. These observations were corroborated by theoretical calculations. Our results demonstrate the quantum-classical crossover in heavy-atom systems. This study establishes a versatile platform for studying a wide range of laser-coolable species.

## INTRODUCTION

Cold collision reactions (1 mK to 1 K) have been an active field in recent years (*1*–*10*). Under such conditions, reactions enter the quantum regime and behave fundamentally differently from the ones at warmer temperatures. First, there are only a few partial waves involved in a cold collision process, so contributions from individual partial waves can be resolved (*1*, *2*, *4*, *6*, *8*). Second, the de Broglie wavelength of the particle becomes comparable to the characteristic interaction length scale, and the reaction is dictated by tunneling and quantum reflection (*11*–*12*). As a result, nonclassical effects, such as shape resonance (*1*, *2*, *4*, *6*) and quantum interference (*6*, *8*, *13*), start to emerge in the cold collision reactions.

Aside from the rich phenomena predicted by the theory, the experimental exploration of reactions in this regime remains challenging due to the difficulty in cooling reactants below 1 K in molecular beam–based experiments. The merged-beam technique with neutral supersonic beams has achieved center-of-mass collision temperatures below 1 K (*1*–*7*). Shape resonance and isotope effects have been observed in the metastable He* + H_2_, HD, D_2_ low-temperature Penning ionization (PI) collisions (*1*, *2*). Chemi-ionization experiments with Ne* further demonstrated quantum-state–controlled channel branching and stereodynamic effects in subkelvin reactive collisions (*4*, *5*). In addition, combining Stark decelerators with merged or crossed beams enables higher-resolution differential cross-sectional (DCS) measurements (*7*, *8*, *14*–*18*). Recent studies on light-atom systems such as H_2_ molecules (*1*, *2*, *8*) and He atoms (*1*, *2*, *13*) have revealed low–partial-wave resonances and interference between partial waves.

On the other hand, laser cooling techniques have enabled the production of ultracold atoms and molecules (*9*, *10*, *19*–*32*), facilitating the study of collisions within optical traps where typically only a single partial wave contributes (*28*–*31*). However, limited by the experimental configuration, it is challenging to control the collision temperature and reach the cold collision regime.

Building upon the principles of laser cooling for preparing cold atoms, we developed a hybrid approach and realized controlled cold collisions between rubidium (^87^Rb) and metastable krypton (^84^Kr*), which hereafter will be referred to as Rb and Kr*, respectively. The collisions between Rb and Kr* atoms result in PI and associative ionization (AI), which are important in atomic and molecular physics, as well as interstellar chemistry (*1*–*5*, *33*). Previous works based on the merged beam technique have studied cold ionization collisions of atoms and molecules in light-atom systems extensively (*1*–*6*). However, observing quantum phenomena in cold collisions of heavy atoms, such as Rb and Kr*, is particularly demanding because achieving a de Broglie wavelength comparable to that of light atoms (He, H_2_, etc.) requires substantially lower temperatures. For instance, to attain a de Broglie wavelength of 1 nm, a rubidium atom must be cooled to nearly 0.1 K, compared to about 5 K for a hydrogen molecule. This temperature requirement, coupled with the need for the angular-resolved product detection, has made the study of cold ionization collision between Rb and Kr* particularly challenging.

To overcome this challenge, we developed an experimental setup that combines laser cooling with the velocity map imaging (VMI) technique (*34*). This configuration enables precise control over the collision temperature and facilitates efficient detection of ion products. With this approach, we achieved the first DCS measurement of the ionization reactions between Rb and Kr* at collision temperatures as low as 15 mK. As the reaction enters the quantum regime, pronounced oscillatory structures are observed in the DCS. Moreover, our experiment reveals angular oscillations in continuum final states alongside a radial oscillatory structure.

## RESULTS

### Experimental setup

**Fig. 1.**
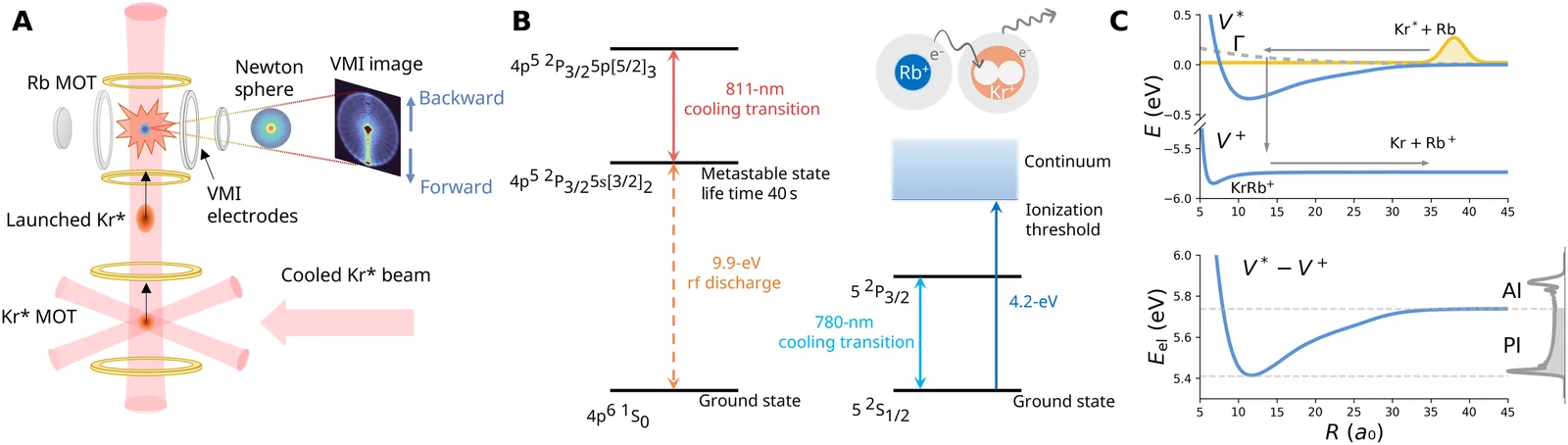
The Kr* + Rb cold collision experiment. (**A**) Experimental setup. Cold Kr* and Rb atoms are prepared in two vertically separated MOTs. Kr* atoms are directed upward via a moving molasses toward the Rb MOT, where collisions occur. The resulting ion products are detected by the VMI system. (**B**) Relevant energy levels of Kr and Rb, including a schematic of the autoionizing (KrRb)* complex. rf, radio frequency. (**C**) Interaction potential curves for the Kr* + Rb and Kr + Rb^+^ pairs. V* and *V* ^+^ are the PECs of the metastable and ionic channels, respectively. Γ is the imaginary part of the complex potential, representing the autoionization width (amplitude exaggerated). The lower curve represents V*-*V* ^+^, representing the possible kinetic energy of the emitted electron. When the kinetic energy of the emitted electron is lower than the asymptotic value of V*-*V^+^*, PI occurs (shaded area in the right inset); otherwise, AI takes place.

The apparatus for studying Kr* + Rb cold collisions is shown in Fig. 1 (*35*). Kr* and Rb atoms are laser cooled (<200 μK) and trapped in two magneto-optical traps (MOTs) positioned 60 cm apart. The lower MOT confines Kr* atoms, whereas the upper MOT contains the target Rb atoms, with its center precisely aligned to a specialized open-geometry VMI system. This configuration allows us to detect ions produced from cold Kr* + Rb collisions and to measure the reaction’s DCS.

The collision energy is controlled by launching the Kr* atoms in a moving molasses (*36*, *37*) toward the Rb cloud, with a certain initial velocity. The launched Kr*(4p^5^5s[3/2]_2_) atoms then undergo cold ionization collisions with the ground-state ^87^Rb atoms (5S_1/2_; *F* = 2). The collision energy is tunable across 15 mK to 8 K, with a typical spread in the center-of-mass frame of 20% at 15 mK and 1% at 8 K (see Materials and Methods and Supplementary Text).

The internal excitation energy of Kr* is 9.9 eV, which exceeds the ionization energy of Rb (4.2 eV). Upon collision, autoionization of Rb by Kr^*^ occurs. Specifically, two ionization processes, namely, PI and AI (*38*, *39*), take place depending on how the released energy is distributed among the reaction products (*38*–*41*)

$$\begin{array}{cc} {\text{Kr}}^{*}+\text{Rb}\to \text{Kr}+{\text{Rb}}^{+}+e^{-} & \left(\text{PI}\right) \end{array}$$ (1)

$$\begin{array}{cc} {\text{Kr}}^{*}+\text{Rb}\to {\text{KrRb}}^{+}+e^{-} & \left(\text{AI}\right) \end{array}$$ (2)

As the electron has a much smaller mass than Kr and Rb atoms, it carries away most of the energy. If the remaining energy exceeds the binding energy of KrRb^+^ ion, then PI occurs. Otherwise, only AI process is allowed. The product ions are focused by the VMI system onto a microchannel plate and detected with a complementary metal-oxide semiconductor camera.

### Theoretical methods

The autoionization processes between Kr* and Rb atoms involve multiple electronic states. During the collision, the Kr* + Rb pair evolves along the lowest electronically excited adiabatic potential energy curve (PEC; Fig. 1A), forming a transient (KrRb)* complex (*38*). This complex can undergo autoionization, releasing an electron and transitioning to the ionic PEC (Kr + Rb^+^; Fig. 1C). The PEC for the metastable state (V*) was computed using the restricted active space self-consistent field (RASSCF) method with spin-orbit coupling (*42*). As shown in Fig. 1C, V* exhibits a potential well with a depth of 0.33 eV (∼2700 cm^−1^) at an internuclear distance of *R* = 11 bohr. The long-range part of the potential was described by an analytical expression to ensure the correct asymptotic *R*^−6^ behavior (*43*). The imaginary component of V* was obtained through a phenomenological fitting with an exponential function. For the ionic curve (*V* ^+^) of Kr + Rb^+^, we adopted an established analytical expression (*44*–*46*), which features a well depth of 0.25 eV (∼2000 cm^−1^) at *R* = 6 bohr. The scattering dynamics results were calculated with a time-independent quantum method for the scattering wave functions on the V* and *V* ^+^ surfaces under the s-wave electron approximation (*47*–*50*). To account for the experimental energy spreading, we applied an energy-dependent convolution to the ion energy distribution. (Further details on the PEC and the dynamics calculations are provided in Materials and Methods and Supplementary Text.)

### Oscillation structure

**Fig. 2.**
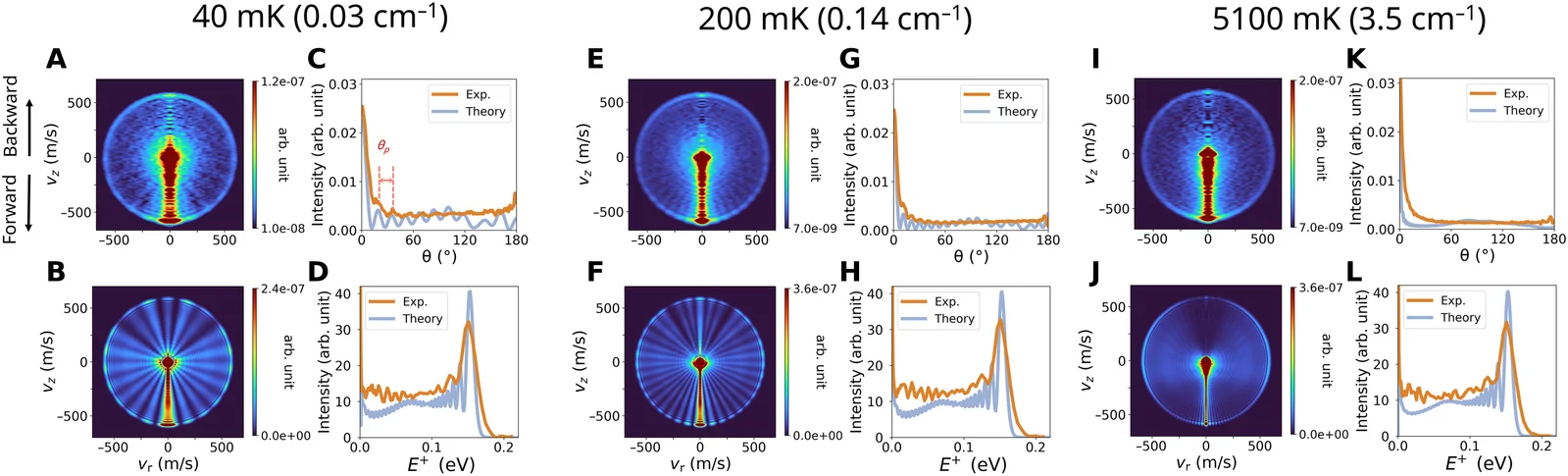
VMI data at 40, 200, and 5100 mK. (**A**, **E**, and **I**) The experimental DCS obtained by inverse Abel transformation of ion signals detected by the VMI [the velocity is relative to the center-of-mass frame, and the arrows next to (A**)** indicate the directions]. arb. unit, arbitrary unit. (**B**, **F**, and **J**) The theoretical velocity distribution obtained by inverse Abel transformation of simulated VMI. (**C**, **G**, and **K**) The experimental and theoretical angular distribution of the ions. θ_p_ in (C) denotes the angular oscillation period. Exp., experiment. (**D**, **H**, and **L**) The experimental and theoretical kinetic energy distribution of the Rb ions.

The experiments were conducted with collision energies ranging from 15 mK to 8 K. The gradual changes of the DCS with the collision energies are displayed in detail in Supplementary Text. Figure 2 shows the result at three representative collision energies: 40, 200, and 5100 mK, respectively. Figure 2 (A, E, and I) is the inverse Abel transformation of VMI signal (see Supplementary Text) (*51*), i.e., the DCS of the reaction. Both angular and radial (energy) distributions of the DCS were extracted (Fig. 2, C, G, and K and D, H, and L). Theoretical calculation results are presented in Fig. 2 (B, F, and J).

We start the discussion from the DCS images. Ion products from both PI and AI reactions are detected in our experiment. To differentiate the KrRb^+^ ions produced in the AI process from the Rb^+^ ions produced in the PI process, we make use of their distinct velocity distributions. In the AI process, the total energy of the system is partitioned between two particles: the KrRb^+^ molecular ion and the ejected electron. Because of energy and momentum conservation, most of the kinetic energy is carried away by the ejected electron. As a result, the velocity of the KrRb^+^ ions is only about 5 m/s in the center-of-mass frame. Therefore, the strong signal near the origin of the image is assigned to the KrRb^+^ ions. Although the velocity distribution of KrRb^+^ ions carries information about the momentum of the ejected electron, the resolution of the current VMI system is insufficient to analyze ions with such low velocities in detail.

The remaining signals correspond to Rb^+^ ions from the PI process. Unlike the AI process, the energy in the PI reaction is distributed among three parties, i.e., Rb^+^, Kr, and electron. Therefore, the velocity distribution of the Rb^+^ ions spans a continuous range from 0 to 620 m/s. In addition, strong forward scattering of Rb^+^ is observed (Fig. 2A). A more detailed inspection reveals oscillatory structures in the radial (energy) and the angular distribution of the Rb^+^ DCS, producing a pattern that resembles a “wagon wheel.” The angular oscillation was found to be periodic, with its period denoted as θ_p_ hereafter (Fig. 2C). These features provide evidence for quantum interference among partial waves in the cold collision process. At 100 mK, the de Broglie wavelength of the Kr atom in the laboratory frame is ∼1.4 nm (27 bohr), which is comparable to the scale of the interaction potential well between the two atoms (Fig. 1C). Under this condition, the discreteness of angular momentum becomes notable, giving rise to the partial-wave interference phenomenon displayed in Fig. 2.

The experimental results are supported by theoretical calculations, which reveal pronounced angular and radial oscillations. The calculated angular oscillation periods closely match the experimental measurements. However, notable differences still exist between the theoretical calculations and the experimental observations. These differences likely stem from the simplified model used in the calculations: (i) the phenomenological fitting of the ionization width as an exponential function (*38*), (ii) dynamics calculations on the lowest spin-orbit–coupled adiabatic PEC only, and (iii) limited accuracy in the shape of the long-range van der Waals potentials. The observed discrepancies highlight the importance of multichannel calculations and accurate ab initio ionization widths, as well as the need for benchmark cold collision experiments to validate different levels of theory.

Figure 2 and the oscillations observed in the DCS therein deserve more detailed discussion. They entail important dynamics of the ionization collision between Kr* and Rb atoms. To understand the mechanism, we studied the scaling of the angular and energy distributions of the DCS with collision energies.

### Angular distribution

**Fig. 3.**
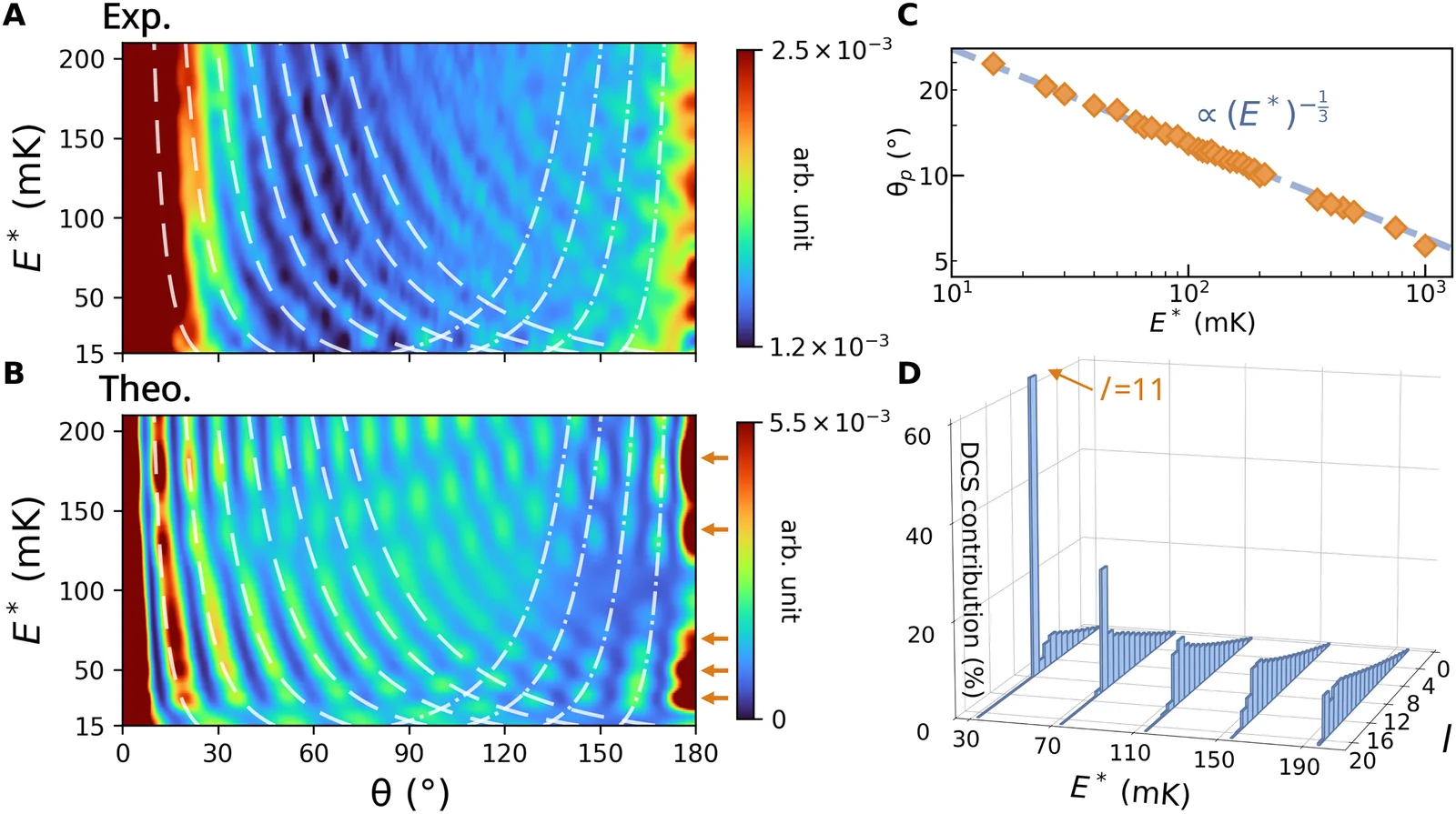
Experimental and theoretical investigation of angular distributions in the DCS. (**A**) Collision energy dependence of the angular DCS. The DCS data for each collision energy were normalized to the total intensity of the VMI signal before inverse Abel transformation. Interpolation was applied between experimental data points. (**B**) Theoretical results. Short white dashed lines denote the −^1^/_3_ power-law dependence of forward oscillation angular period on collision energy (fitted from Fig. 3C data), whereas white dot dashed lines indicate the backward oscillation period. The arrows indicate the collision energies that shape resonances occur. Theo., theory. (**C**) In the 15-mK to 1-K collision energy range, the angular period of forward scattering oscillation follows the −^1^/_3_ power scaling. Error bars are within the data points. (**D**) Calculated contribution of individual partial waves at various collision energies. At E* = 32 mK, the contribution from the highest partial wave (*l* = 11) is enhanced substantially, which indicates a shape resonance (the column pointed by the arrow).

When the collision temperature exceeds 200 mK, the angular oscillation structures become blurred due to the combined effects of averaging from more participating partial waves and the limited resolution of the VMI system. Below 200 mK, however, the DCS exhibits pronounced oscillatory structures. To reveal the dependence on collision temperatures, we performed finer scans of the DCS with an energy resolution of ∼10 mK to obtain the “Angular distribution-collision Temperature” spectrum (A-T spectrum) as shown in Fig. 3A. Well-defined angular oscillations appear in both forward and backward directions within the 15- to 210-mK range. Although they share the same periodicity, the backward angular oscillations have a lower contrast compared to their forward counterparts. Theoretical results in Fig. 3B show similar angular oscillation structures and are in qualitative agreement with the experimental observations. We observed that the period of the angular oscillation decreases with increasing collision temperature. Figure 3C shows the oscillation period plotted against collision energy in the range of 15 mK to 1 K, revealing a −^1^/_3_ power-law scaling that holds up to a collision energy of 1 K. This scaling likely extends beyond 1 K. However, the limited experimental resolution prevents clear resolution of the finer angular oscillations at higher energies. Meanwhile, as the temperature continues to rise, the number of participating partial waves also increases progressively, causing the angular oscillations to gradually vanish because of averaging effects, marking a transition from quantum to classical regime.

The observed −^1^/_3_ power-law scaling is different from the diffraction oscillations observed in inelastic collisions, which exhibit a −^1^/_2_ power-law scaling with collision energy predicted by the hard-sphere models (*8*). This behavior provides evidence that we were probing the long-range van der Waals potentials in the cold collision experiment. It can be understood within the framework of the Langevin capture model (*52*). This model is often used to estimate the total scattering cross section, but here, it is applied to estimate the DCS for partial waves. At low collision temperature, the number of partial waves participating in the collision decreases, allowing the atom to probe the long-range interaction between Kr* and Rb, which follows a *C*_6_/*R*^6^ form (*11*–*12*). Under these conditions, the reaction has a highest partial wave, *l*_max_, which is determined by the kinetic energy of the incident Kr* atom and the centrifugal barrier of the collision (see Materials and Methods). For the long-range interaction of a *C*_6_/*R*^6^ form, the angular momentum of the highest partial wave *l*_max_ is related to the collision energy (or temperature) $E^{*}$ as $l_{\text{max}}=2^{-1/3}\sqrt{6\mu /\hbar^{2}}\;C_{6}^{1/6}{\left(E^{*}\right)}^{1/3}$.Theoretical calculations reveal a sharp decline in the partial-wave DCS, i.e., the contribution of individual partial waves, near *l*_max_ (Fig. 3D), further corroborating this picture.

In nonresonant cases, adjacent partial waves near *l*_max_ have similar phase shifts. Their superposition, with proximate phase but different *l* values, leads to the enhanced forward scattering. To analyze the angular oscillation, we incorporated the combined contributions from the partial waves with *l < l*_max_. The asymptotic properties of the Legendre function $P_{l}\left(\text{cos}\;\theta \right)$ in the range of 1*/l <* θ *< l* can be used, where θ is the polar angle. Asymptotic analysis then indicates that the angular oscillation period is governed by the sharp cutoff at *l*_max_, yielding a semiclassical result $\theta_{p}\approx \pi /l_{\text{max}}\propto {\left(E^{*}\right)}^{-1/3}$ (see Materials and Methods). This behavior is reproduced by theoretical calculations (Fig. 3B). Although the Langevin model (a classical model) is applied, the angular oscillations in the DCS are fundamentally quantum and have no classical counterpart.

**Fig. 4.**
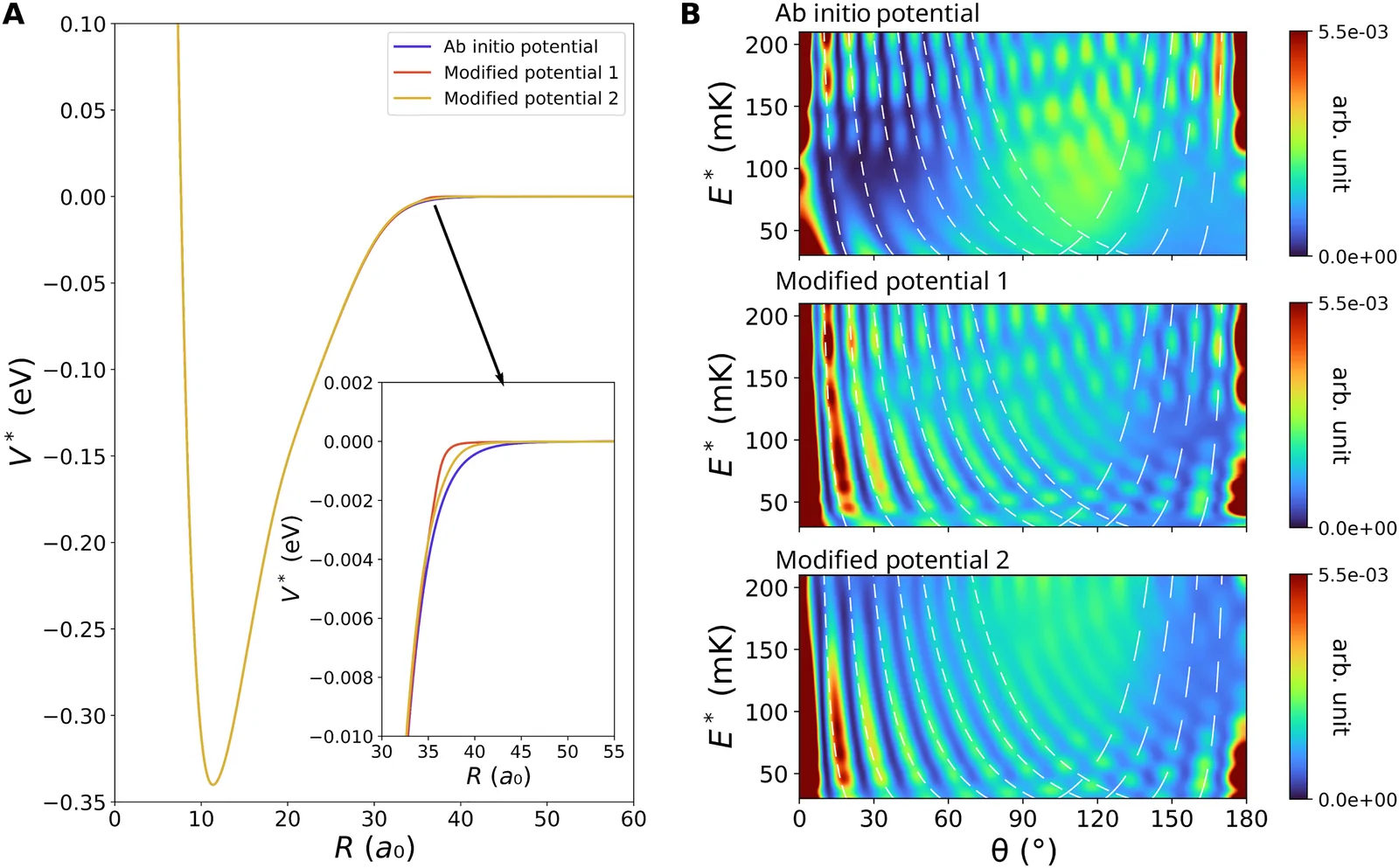
The sensitivity of the A-T spectrum to the Kr* + Rb PEC. (**A**) Ab initio PEC for the entrance channel along with two modified potential curves. (**B**) Angular DCS calculated from the ab initio potential (top) and from the two modified potentials (middle and bottom). The “modified potential 1” (same as Fig. 3B) shows better agreement with the experimental results.

In addition to the scaling of the angular oscillations period with the collision temperature, a closer examination of the A-T spectrum reveals further information about the cold collision process. We found that the interference patterns are extremely sensitive to small changes in the long-range part (35 to 45 bohr) of the Kr* + Rb PEC (see Fig. 4 and Supplementary Text). Consequently, the A-T spectrum provides more stringent constraints for theoretical calculations than does a single DCS measured at a fixed collision energy. Another noteworthy feature observed in the A-T spectrum is the enhanced backward scattering (around 180°), whose magnitude is modulated by the collision energy in the range of 15 to 210 mK. The enhancement peaks at specific energies of ∼40, 70, 120, and 170 mK. Theoretical calculations also reveal a similar phenomenon, although the peak locations and amplitudes do not exactly match the experimental results. At the collision temperatures where backward scattering peaks (32, 50, 70, 135, and 178 mK), the calculations indicate the presence of shape resonances. At these collision energies, the reaction cross section is dominated by a specific partial wave when the incident energy matches the energy of a quasibound state behind the centrifugal barrier. For instance, at 32 mK, the theoretical calculations find a clear resonance in the *l* = 11 partial wave (see Fig. 3D). By contrast, at nonresonant energies, the angular distribution exhibits less backward enhancement due to destructive interference between the S-matrix elements (see Materials and Methods). The theoretical calculations also predicted resonance peaks on the total cross section at these collision temperatures, whereas the experimentally measured total cross section lacks such resonant features.

The observed discrepancies between theory and experiment likely arise from the use of a simplified single-channel model in the calculations, whereas the Kr* + Rb collision is intrinsically multichannel when electron spins, spin-dependent interactions, etc., are considered. If a resonance is localized within a specific channel, then nonresonant scattering from other channels could obscure the resonance signature. Moreover, interchannel coupling implies that a single-channel model is inadequate for accurately characterizing the resonance phenomena in this system. Last, the high sensitivity of cold collision processes to the long-range potential makes the precise prediction of resonance energies exceptionally challenging (see Supplementary Text). All in all, these underscore the need to develop more sophisticated multichannel theoretical models in the future. They also demonstrate that the A-T spectrum is more sensitive to the scattering resonances and can help uncover subtle resonances that are difficult to detect in total cross-sectional measurements.

### Energy distribution of the Rb^+^ ions

**Fig. 5.**
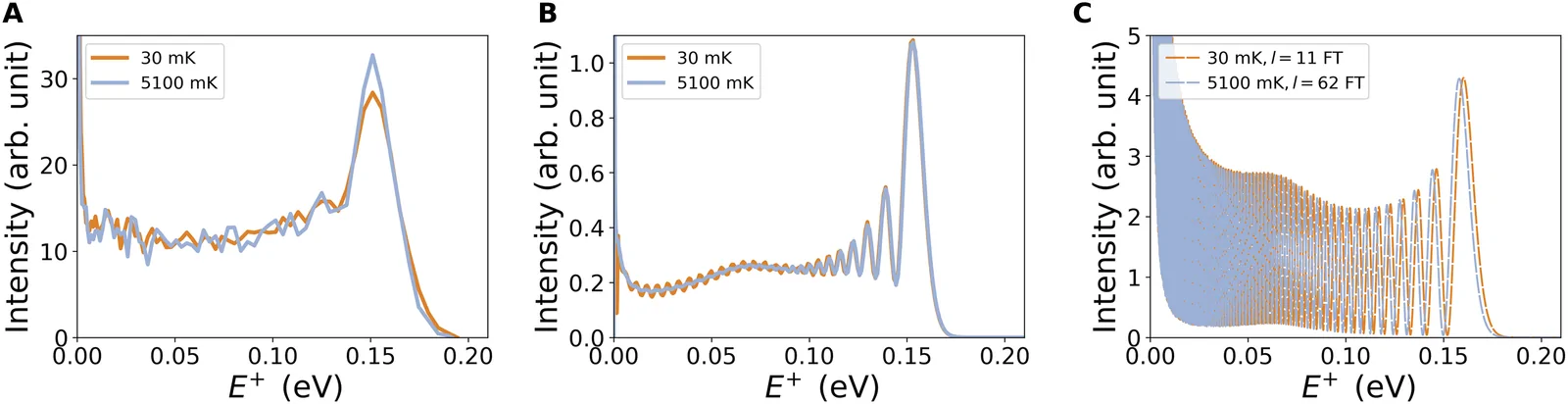
Energy distribution of ions. (**A**) Experimentally observed kinetic energy distributions of Rb^+^ at collision energy of 30 mK (orange) and 5100 mK (blue). The distributions are normalized for comparison. (**B**) Simulated normalized kinetic energy distributions of Rb^+^ at the same collision energies. (**C**) The Fourier analysis of $\sqrt{\Gamma }{\psi_{l}}^{*}$, where 𝑙 is the partial wave that contributes the most at the given collision energy. The squared magnitudes of the Fourier transforms are plotted as functions of the exit energy *E^+^*. FT, Fourier transform.

For the radial structures in the DCS, the normalized kinetic energy distributions of Rb^+^ at different energies are plotted in Fig. 5A. These distributions exhibit similar oscillatory structures and show little variation with collision energy. The cutoff energy near 0.16 eV is mainly determined by the well depth of the Kr* + Rb PEC (*40*), which limits the maximal energy that can be distributed among the collision products. The oscillatory structure of the energy distribution originates from oscillations in the autoionization transition matrix element. Because the potential well of the Kr* + Rb interaction curve lies at a relatively large internuclear distance, corresponding to a flat region of the ionic PEC *V* ^+^, the final-state wave function on the ionic curve can be approximated as asymptotic plane waves. Thus, given the scattering wave function $\psi^{*}$ of the Kr* + Rb PEC, the autoionization transition matrix elements can be viewed as the Fourier transformation of $\sqrt{\Gamma }\psi^{*}$ from coordinate space to momentum space (*53*), modulated by a fast-oscillating cosine term (see Materials and Methods). In Fig. 5C, the normalized momentum distribution of $\sqrt{\Gamma }\psi^{*}$ exhibits nearly identical spectral profiles to the DCS as a function of exit energy, confirming that it indeed governs the energy distribution of the DCS. Regarding the overall envelope of the energy distribution of the DCS, it is controlled by the shape of the wave function in the short range. Given that the depth of potential well is much larger than the low collision energy, the wave functions are expected to have similar shapes across different incident collision energies. Although the long-range potential and incident energy influence the overall amplitude of the wave function, they have little effect on its shape in the short-range region. Consequently, normalized ion kinetic energy spectra remain nearly independent of the incident energy. This picture is supported by theoretical calculations, which reproduce the cutoff energy and kinetic energy distributions of Rb^+^ in close agreement with experimental observations (Fig. 5, B and C).

## DISCUSSION

In summary, we achieved cold collision between Kr* and Rb and measured the DCS of the Kr* + Rb ionization reaction, with collision temperatures as low as 15 mK. The combination of laser cooling and VMI technology enabled us to control the collision temperature over two orders of magnitude, allowing the experiment to transit from the classical regime into the quantum regime. At low collision energies, oscillatory structures in both radial and angular distributions on the DCS were observed, revealing distinct signatures of quantum scattering. Although theoretical calculations successfully reproduced the main experimental observations, a complete understanding of the remaining discrepancies will require the future development of a multichannel theoretical model for this reaction.

Aside from the need for additional theoretical efforts, the versatility of the setup opens the door to numerous near-future experimental studies. These range from studies of stereodynamics using spin-polarized atoms to evaluations of different reaction channels’ contributions and explorations of quantum control in cold reactive collisions.

Given that metastable rare gas, alkali metal, and alkaline earth metal atoms can all be laser cooled (*54*), this experimental method can be readily extended to similar collision systems (e.g., He* + Sr). Furthermore, with the advent of laser cooling technology for molecules (*55*), cold molecules can also be introduced in the experimental configuration reported in this work. With the increased complexity in such systems, we anticipate even richer physical and chemical phenomena.

## MATERIALS AND METHODS

### Experiment

The experimental setup is illustrated in Fig. 1. Initially, we excited Kr atoms from the ground state 4p^6^ S_0_ to the metastable state 4p^5^ 5s[3/2]_2_, which has an energy of 9.9 eV, using a radiofrequency discharge. Laser cooling and atom manipulation were carried out on the transition between these two states at 811 nm. The divergent beam of Kr* atoms was then collimated and focused through a transverse cooling stage and a two-dimensional MOT to enhance its intensity. Subsequently, the atoms were decelerated in a Zeeman slower and loaded into the lower MOT, accumulating approximately 5 × 10^7^ atoms.

To launch the Kr* atoms, we used the moving molasses technique. The MOT magnetic field was first switched off, and the frequency detuning of the two vertical MOT beams was rapidly ramped to ±∆δ relative to their original values. This frequency shift imparts an initial velocity to the atomic cloud given by the relation *v* = ∆δ/*k*. Last, the intensities and detuning of all MOT beams were adjusted to implement Sisyphus cooling across the entire cloud. This process reduces the temperature and limits spatial expansion during launch. Using absorption imaging method, the temperature of the launched Kr* cloud was measured to be 50 μK.

The Rb MOT in the science chamber is loaded from a separate source MOT. It contains approximately 5 × 10^7^ Rb atoms. To ensure that all Rb atoms are in the ground state (5S_1/2_) before the Kr* + Rb collision, the cooling light for the Rb MOT is switched off 1 ms in advance. The temperature of the released Rb cloud was measured to be around 170 μK. The detailed timing sequence is provided in Supplementary Text. The experiment operates at a repetition rate of 1 Hz.

The primary background in the experiment originated from ion signals produced by Kr* + Kr* collisions within the launched Kr* cloud, which were approximately uniformly distributed. Before conducting the Kr* + Rb collision experiments, the background signal was measured and smoothed to eliminate noise. This smoothed background was then subtracted during subsequent Kr* + Rb collision measurements. In each experimental cycle, Kr* + Rb collisions generated tens of ions. The experiment was repeated multiple times to accumulate sufficient signal and achieve the desired signal-to-noise ratio.

### Potential energy curves

#### Kr*Rb PECs

In treating the electronic states of Kr* + Rb penning ionization system, a principal difficulty arises because Kr*Rb states are submerged in the continuum of states of the (KrRb)^+^ + e^−^ products. The electronic wave function of the latter cannot be correctly described by standard electronic structure methods designed for the description of bound states, and the variational principle is expected to drive Kr*Rb states down either to the ground state or one of the excited bound states. Therefore, during the wave function optimization, one should seek for a constraint on the electron configurations to keep consistent with Kr*Rb states. To this end, the RASSCF method was adopted in this work. In the RASSCF calculation, the 5s orbitals of both Rb and Kr were kept singly occupied, and 1,2 ^2^ Σ, 1,2 ^2^ $\Pi_{x}$, 1,2 ^2^ $\Pi_{y}$, ^4^ Σ, ^4^ $\Pi_{x}$, and ^4^ $\Pi_{y}$ were averaged, each of which corresponds to the electron configuration where one 4p orbital of Kr was singly occupied. The obtained wave functions are eigenfunctions of the spin-free electronic Hamiltonian ($H_{\text{SF}}$). Once these states are determined, the spin-orbit couplings ($H_{\text{SO}}$) between these states can be readily obtained. Then, the eigenenergies of total electronic Hamiltonian ($H^{e}=H_{\text{SF}}+H_{\text{SO}}$) will give a detailed description of the PECs of Kr* and Rb.

**Fig. 6.**
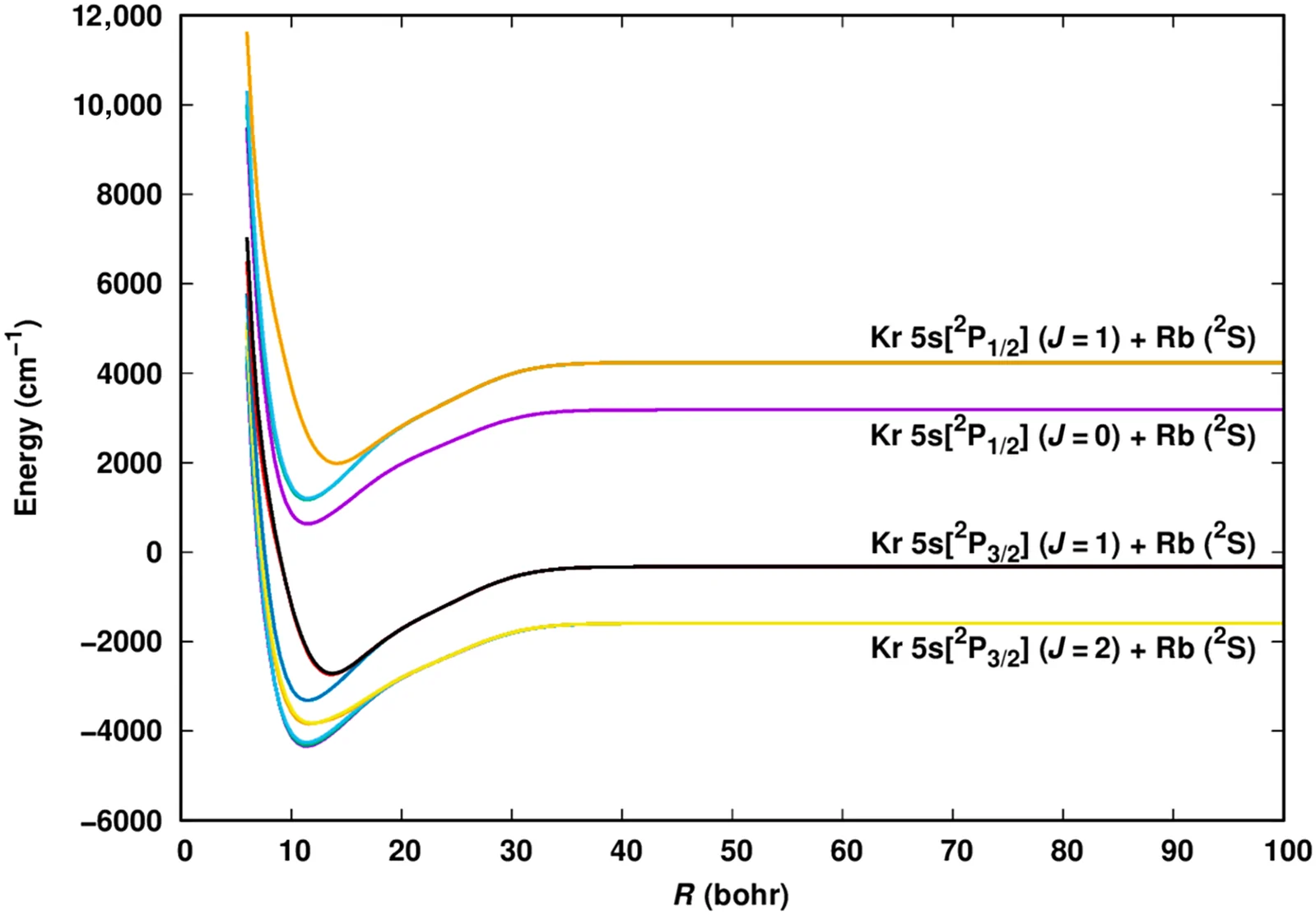
PECs of Kr*Rb with spin-orbit splitting.

As can be seen from Fig. 6, when Kr* and Rb are separated by a large distance, the PECs can be classified into four groups with degeneracies of 5, 3, 1, and 3, which are consistent with the splitting of Kr* energy levels. The relative energies of degenerate groups are 0.00, 1267.17, 4774.79, and 5827.09 cm^−1^, respectively, which are in good agreement with the experimentally determined splittings (0.00, 945.026, 5219.87, and 5874.96 cm^−1^), thus verifying the validity of RASSCF wave functions.

#### KrRb^+^ PECs

The electronic structure of KrRb^+^ is relatively simple, and a large amount of work has been undertaken on the interaction of alkali-metal cations (A^+^) with a rare gas partner (Rg). In this work, the following potential function is chosen to model the KrRb^+^ PEC

$$\begin{array}{cc} V\left(R\right)= & -\frac{\alpha_{\text{RgD}}Z^{2}}{2R^{4}}-\frac{C_{6}}{R^{6}}-\frac{\alpha_{\text{RgQ}}Z^{2}}{2R^{6}}+\frac{B_{\text{Rg}}Z^{3}}{2R^{7}}\\ & -\frac{C_{8}}{R^{8}}-\frac{\alpha_{\text{RgO}}Z^{2}}{2R^{8}}-\frac{\gamma Z^{4}}{24R^{8}}+Ae^{-\mathrm{bR}} \end{array}$$ (3)

where *Z* is the “effective” charge on the A^+^ ion; $\alpha_{\text{RgD}}$, $\alpha_{\text{RgQ}}$, and $\alpha_{\text{RgO}}$ are the dipole, quadrupole, and octupole polarizabilities of the Rg atom; $B_{\text{Rg}}$ is the higher-order “dipole-quadrupole” polarizability of the Rg atom; γ is the higher-order “second dipole hyperpolarizability” of the Rg atom; and $C_{6}$ and $C_{8}$ are *Z*-independent coefficients representing the first and second terms in the dispersion interaction. More details of PEC construction are provided in Supplementary Text.

### Langevin model of the collision

In the Langevin model, a reaction can occur only if the kinetic energy of the incident particle exceeds the centrifugal barrier. This result is often used to estimate the total scattering cross section, but it can also be applied to estimate the DCS for partial waves. In classical scattering, given the collision energy $E^{*}$ and angular momentum *L*, the impact parameter *b* can be expressed as $\frac{L}{\sqrt{2\mu E^{*}}}$, where μ is the reduced mass. For neutral atomic systems governed by van der Waals long-range interactions, particles incident with an impact parameter less than $b_{\text{max}}=\frac{\sqrt{3}}{2^{1/3}}{\left(\frac{C_{6}}{E^{*}}\right)}^{1/6}$ have equal probability P of undergoing a reaction in the Langevin capture model. Therefore, the DCS with respect to b can be expressed as the following piecewise function

$$\sigma \left(b,E^{*}\right)db=\left\{ \begin{array}{ll} P\;d\left(\pi b^{2}\right)=2\pi \mathrm{bP}db, & b<b_{\text{max}}\\ 0, & b>b_{\text{max}} \end{array}\right.$$ (4)

where *P* is the reaction probability. Here, $b_{\text{max}}$ corresponds to the angular momentum

$$L_{\text{max}}=2^{-1/3}\sqrt{6\mu }C_{6}^{1/6}{\left(E^{*}\right)}^{1/3}$$ (5)

The expression can thus be transformed into the DCS with respect to angular momentum (*56*)

$$\sigma \left(L,E^{*}\right)dL=\left\{ \begin{array}{ll} P\frac{\pi L}{\mu E^{*}}dL, & L<L_{\text{max}}\\ 0, & L>L_{\text{max}} \end{array}\right.$$ (6)

**Fig. 7.**
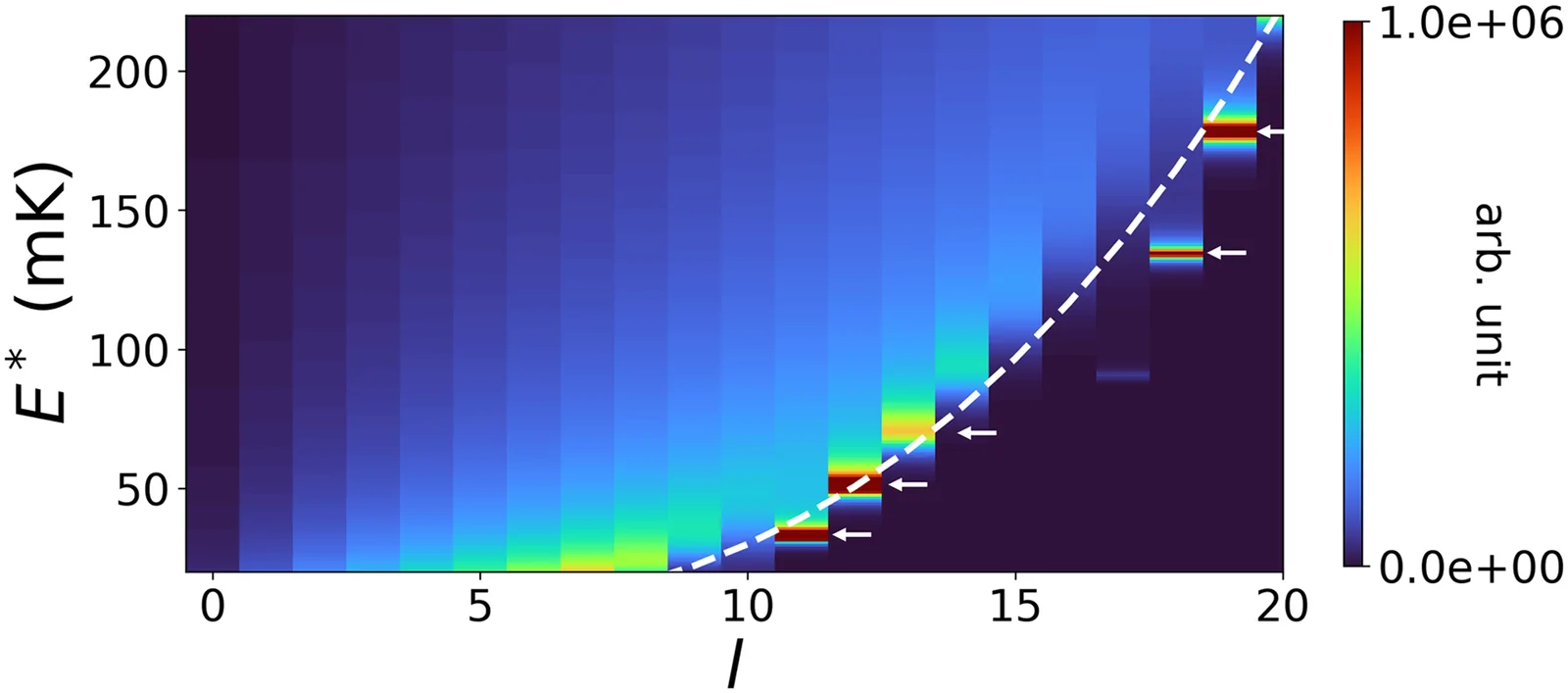
Relative magnitudes of partial wave DCS under different incident collision energies $E^{*}$ and corresponding angular momentum quantum numbers l. The white dashed lines indicate the maximum of the potential barrier $V_{b}$ in the effective potential $V_{l}^{*}=V^{*}+l\left(l+1\right)/\left(2\mu R^{2}\right)$.

In quantum scattering, the angular momentum *L* is quantized as $\sqrt{l\left(l+1\right)}$ ℏ ≈ *l*ℏ, where *l* is the angular momentum quantum number of each partial wave. Therefore, the capture model predicts that the DCS for each partial wave takes the form

$$\sigma_{l}\left(E^{*}\right)\left\{ \begin{array}{ll} \propto l/E^{*}, & l<l_{\text{max}}\\ =0, & l>l_{\text{max}} \end{array}\right.$$ (7)

for nonresonant cases (Figs. 3D and 7 and fig. S13A). A more detailed analysis presented below reveals that the oscillatory pattern in the angular distribution is primarily governed by the sharp cutoff at *l*_max_.

Recalling the asymptotic form of the Legendre polynomial valid for 1/l<θ<1, $P_{l}\left(\text{cos}\theta \right)∼\text{sin}\left[\left(l+1/2\right)\theta +\pi /4\right]/\sqrt{l\;\text{sin}\theta }$, we can evaluate the scattering amplitude as

$$f\left(\theta \right)\propto \sum_{l=0}^{l_{\text{max}}}\left(l+\frac{1}{2}\right)e^{i\;\eta_{l}}P_{l}\left(\text{cos}\theta \right)\propto \frac{1}{\sqrt{\;\text{sin}\theta }}\sum_{l=0}^{l_{\text{max}}}\frac{l+\frac{1}{2}}{\sqrt{l}}e^{i\;\eta_{l}}\;\text{sin}\left[\left(l+\frac{1}{2}\right)\theta +\frac{\pi }{4}\right]$$ (8)

where $\eta_{l}$ is the phase of the S-matrix element (see Eq. 11 in the following section). $\eta_{l}+\pi l$ typically varies slowly below $l_{\text{max}}$ (up to a multiple of 2π) (fig. S14). Assuming $l_{\text{max}}$ ≫ 1, $1/l_{\text{max}}\ll \theta <1$, and $\eta_{l}+\pi l$ is slow varying compared to θl, the summation can be further approximated by an integral. The asymptotic form of this integral can be obtained via integration by parts and by discarding the integral of the high-frequency oscillatory terms (*57*)

$$\begin{array}{c} \frac{1}{\sqrt{\text{sin}\theta }}\sum_{l=0}^{l_{\text{max}}}\frac{l+\frac{1}{2}}{\sqrt{l}}e^{i\;\eta_{l}}\;\text{sin}\left[\left(l+\frac{1}{2}\right)\theta +\frac{\pi }{4}\right]\\ ∼\frac{1}{\sqrt{\text{sin}\theta }}\int_{0}^{l_{\text{max}}}dl\;\sqrt{l}e^{i\;\eta \left(l\right)+i\;\pi l}\;e^{-i\;\pi l}\text{sin}\left[\left(l+1/2\right)\theta +\frac{\pi }{4}\right]\\ ∼\frac{1}{-\theta^{2}+\pi^{2}}e^{i\;\eta \left(l_{\text{max}}\right)}\sqrt{\frac{l_{\text{max}}}{\text{sin}\theta }}\left\{i\pi \;\text{sin}\left[\left(l_{\text{max}}+1/2\right)\theta +\frac{\pi }{4}\right]\right.\\ \left.+\text{θcos}\left[\left(l_{\text{max}}+1/2\right)\theta +\frac{\pi }{4}\right]\right\} \end{array}$$ (9)

Therefore, as $l_{\text{max}}$ remains far larger than 1, the angular oscillation period can be approximated as follows

$$\theta_{p}\approx \frac{\pi }{l_{\text{max}}}=\left(\frac{2^{1/3}\pi \hbar }{\sqrt{6\mu }}C_{6}^{-1/6}\right){\left(E^{*}\right)}^{-1/3}$$ (10)

This formula can be used to estimate the *C*_6_ coefficient from experimental data. This *C*_6_ was used as the initial value in the more rigorous quantum dynamic calculations to get the angular oscillations. This resulted in a θ_p_ approximately 1.1 times larger than the experimental observation. This difference may arise from the limitation of the Langevin model or from the underestimation of ∆ $(\eta_{l}+\pi l)/∆l$. Then, the *C*_6_ was adjusted until θ_p_ matched the experiments (Fig. 3B). The final value of *C*_6_ is about 1.8 (i.e., ∼1.1^6^) times of the initial estimation.

### Scattering dynamics calculations

The autoionization process can occur during collisions between metastable Kr* atoms and ground-state Rb atoms, leading to ion formation via the PI reaction

$$Kr^{*}+\text{Rb}\to \text{Kr}+{\text{Rb}}^{+}+e^{-}$$ (11)

In this framework, ionization is treated as a Franck-Condon–type transition. The nuclear motion in the entrance channel is described by a local complex potential, with the imaginary part representing the loss of population due to ionization. At low collision energies and relatively high electron energies, the ionization process can also be described within the Born-Oppenheimer approximation. The radial nuclear wave functions of the ${\text{Kr}}^{*}+\text{Rb}$ entrance channel, $\psi^{*}\left(R\right)$, are obtained from the following time-independent Schrödinger equation (*47*)

$$\left[\frac{1}{2\mu }\frac{d^{2}}{{dR}^{2}}+\frac{l^{*}\left(l^{*}+1\right)}{2\mu R^{2}}+V^{*}\left(R\right)-\frac{i}{2}\Gamma \left(R\right)\right]\psi^{*}\left(R\right)=E^{*}\psi^{*}\left(R\right)$$ (12)

where μ is the reduced mass, R is the interatomic distance, and $l^{*}$ is the angular momentum. $V^{*}\left(R\right)$ is the adiabatic spin-orbit–corrected potential energy of metastable channel, Γ(R) is the imaginary part of the complex potential, which represents the autoionization width, and $E^{*}$ is the collision energy in the entrance channel. Similarly, the radial nuclear wave functions of the $\text{Kr}+Rb^{+}$ ionic exit channel are obtained from a similar Schrödinger equation

$$\left[\frac{1}{2\mu }\frac{d^{2}}{dR^{2}}+\frac{l^{+}\left(l^{+}+1\right)}{2\mu R^{2}}+V^{+}\left(R\right)\right]\psi^{+}\left(R\right)=E^{+}\psi^{+}\left(R\right)$$ (13)

where $V^{+}\left(R\right)$ is the adiabatic potential energy of the ionic channel.

Under the s-wave electron approximation (where the Penning electron angular momentum λ=0), the angular momentum number of each partial wave is conserved (*48*–*50*), i.e., $\Delta \;l=l^{*}-l^{+}=0$. The energy distribution of the ejected electron energy ε is given by (*47*, *48*, *58*)

$$\frac{d\sigma_{v}\left(E^{*}\right)}{\mathrm{d\varepsilon}}=g^{*}\frac{2\;\pi^{2}}{{k^{*}}^{2}}\sum_{l}\left(2l+1\right){|\langle {\left(\psi_{l}^{+}\right)}^{o}|\sqrt{\Gamma }|{\left(\psi_{l}^{*}\right)}^{i}\rangle |}^{2}$$ (14)

where ε is the energy of an ejected electron; $k^{*}$ is the asymptotic wave vector of the entrance channel; superscripts “*o*” and “*i*” of wave functions indicate outgoing and incoming scattered waves, respectively; and $g^{*}$ is the statistical weight of the autoionizing entrance channel. For PI processes, the total energy is given by $E_{\text{tot}}=E^{*}+E_{\Delta }=E^{+}+\varepsilon$, where $E_{\Delta }$ is the energy difference between the two asymptotes. Thus, the energy distribution of ejected ions $f\left(E^{+}\right)$ can be described using the aforementioned formula for electron energy spectra.

By integration over scattering angles, the cross-sectional formula can be expressed as (*50*)

I(E+,θ)=14k2∣∑l=0(2l+1)⟨(ψl+)o∣Γ∣(ψl∗)i⟩Pl(cosθ)∣2 (15)

where $k^{2}=2\mu E^{*}$ and $P_{l}$ is the Legendre polynomials.

The radial nuclear wave functions are calculated with the Numerov method (*59*), and the autoionization matrix elements (i.e., the S-matrix elements) $\left\langle \psi^{+}|\sqrt{\Gamma }|\psi^{*}\right\rangle$ are then evaluated before calculating the ion energy distribution. In the calculations, the parameter $g^{*}$ is set to 1.

### Transition matrix element oscillations

Because the potential well of the Kr* + Rb interaction curve is located at an internuclear distance range relatively outward, corresponding to a flat region of the ionic PEC *V*^+^, the final state wave function on *V*^+^ can be treated as asymptotic plane waves. As a result, the autoionization transition matrix elements can be approximated as follows

$$\begin{array}{cc} S\left(E^{+}\right) & =\int_{0}^{\infty }dR\;\psi_{E^{+}}^{+}\;\sqrt{\Gamma }\;\psi^{*}\approx \frac{1}{\sqrt{k_{\epsilon }}}\int_{0}^{\infty }dR\;\text{cos}\left(k_{E^{+}}R-\frac{1}{2}\pi l+\eta_{E^{+},l}\right)\sqrt{\Gamma }\left(R\right)\psi^{*}\left(R\right)\\ & =\frac{1}{\sqrt{k_{E^{+}}}}\text{Re}\left\{\text{exp}\left[i\left(-\frac{1}{2}\pi l+\eta_{E^{+},l}\right)\right]\int_{-\infty }^{\infty }dR\;e^{i\;k_{E^{+}}R}\sqrt{\Gamma }\left(R\right)\psi^{*}\left(R\right)\right\} \end{array}$$ (16)

An even extension of $\sqrt{\Gamma }\psi^{*}$ is used. The phase factor $\text{exp}\left[i\left(-\frac{1}{2}\pi l+\eta_{E^{+},l}\right)\right]$ arises from the scattering phase shift of the ionic PEC and contributes to the rapid oscillations in $\frac{d\sigma_{v}\left(E\right)}{\mathrm{d\varepsilon}}$. Because these oscillations are smoothed out by the limited experimental resolution, the slowly varying part of ${|S\left(E^{+}\right)|}^{2}$ can be captured by $\int_{-\infty }^{\infty }dR\;e^{i\;k_{E^{+}}R}\sqrt{\Gamma }\left(R\right)\psi^{*}\left(R\right)$, i.e., the Fourier transformation of $\sqrt{\Gamma }\psi^{*}$. Taking into account the experimental resolution $\rho \left(E^{+},\epsilon \right)$, the observed intensity in the exit energy interval $E^{+}∼E^{+}+\Delta E^{+}$ can be expressed as

$$\begin{array}{c} \Delta E^{+}\int \mathrm{d\epsilon}\;{|S\left(E^{+}+\epsilon \right)|}^{2}\rho \left(E^{+},\epsilon \right)\approx \frac{\Delta E^{+}}{k_{E^{+}}}{\left|\int_{-\infty }^{\infty }dR\;e^{i\;k_{E^{+}}R}\sqrt{\Gamma }\left(R\right)\psi^{*}\left(R\right)\right|}^{2}\\ \propto {\left|\int_{-\infty }^{\infty }dR\;e^{i\;k_{E^{+}}R}\sqrt{\Gamma }\left(R\right)\psi^{*}\left(R\right)\right|}^{2}\Delta k_{E^{+}} \end{array}$$ (17)

### Scattering resonances

We analyze the partial-wave–resolved behavior of the system. As shown in Fig. 7, the relative magnitudes of the DCS for different partial waves are plotted as functions of collision energy (temperature). The white line indicates the barrier heights $V_{b}$ of the effective potential $V_{l}^{*}$ for each partial wave. These barrier heights act as critical thresholds dividing the figure (Fig. 7) into two distinct regimes: $E^{*}>V_{b}$ (right side of the dashed line in fig. S17) and $E^{*}<V_{b}$ (left side of the dashed line in Fig. 7).

When $E^{*}>V_{b}$, the wave function can surmount the barrier and penetrate into the small internuclear distance region. This allows efficient vertical transitions mediated by the autoionization width, leading to enhanced ionization. In contrast, for $E^{*}<V_{b}$, substantial wave function amplitude in the short-range region arises only in the vicinity of some discrete collision energies via shape resonances, which enhance the magnitude of short-range wave function and thus the ionization probability. Notably, relatively broad resonance peaks may still be present even when the collision energy is slightly above the potential barrier, as exemplified by the *l* = 13 partial wave at about 70 mK in Fig. 7. Their origin is attributed to quantum reflection by the centrifugal barrier, as opposed to tunneling (*1*, *6*). The inherent energy broadening of the incident Kr* atom cloud may partially obscures these resonant quantum tunneling effects in the experiment.

**Fig. 8.**
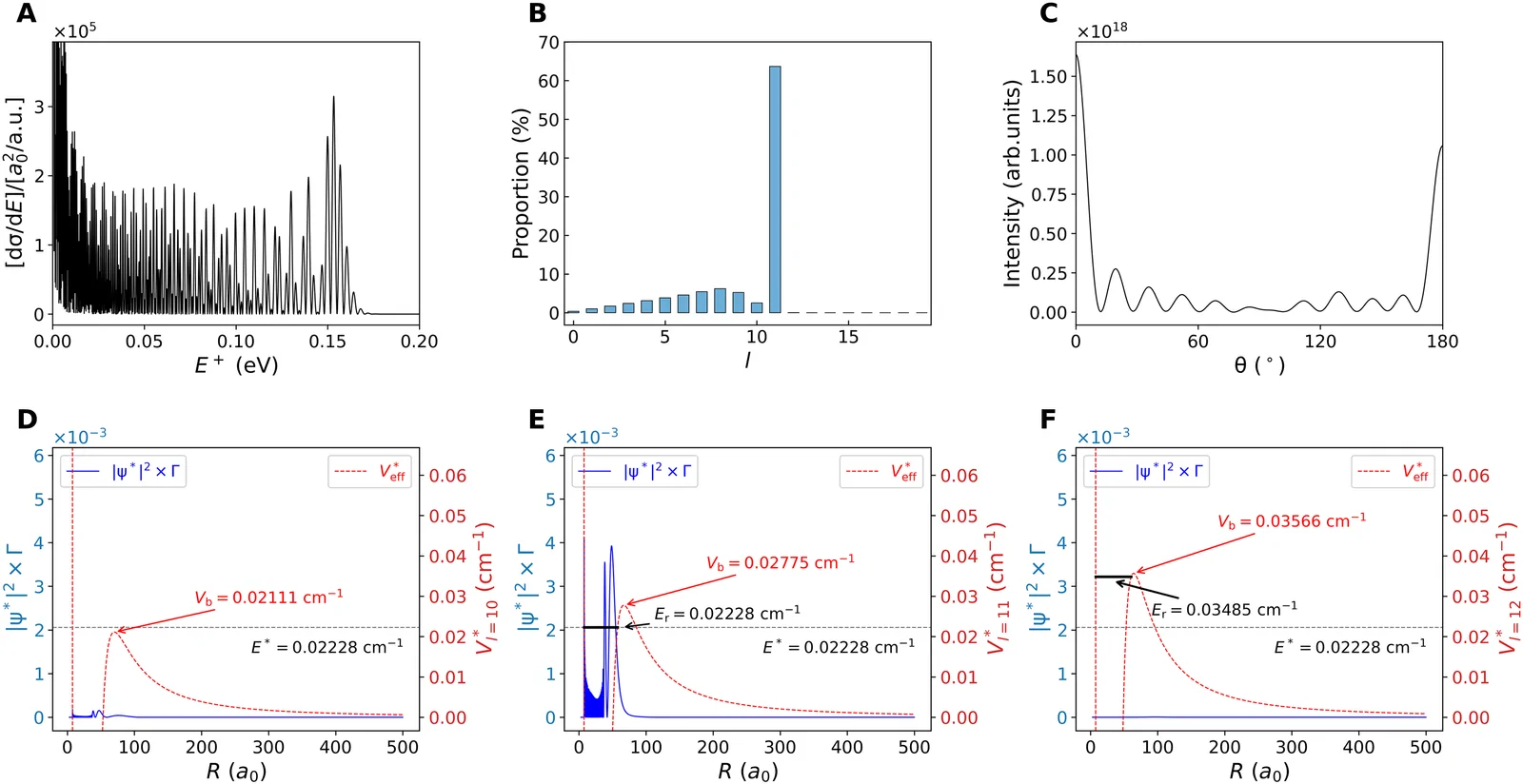
Cold collision at the temperature with shape resonance. (**A**) The ion energy distribution of Kr* + Rb penning ionization. a.u., arbitrary unit. (**B**) Relative contribution of single partial-wave DCS to the ICS (normalized to 100%). (**C**) Angular DCS. (**D** to **F**) The effective potential $V_{l}^{*}$ (red dashed curve, barrier height $V_{b}$); squared modulus of the wave function multiplied by the autoionization width ${|\psi^{*}|}^{2}\times \Gamma$ (blue solid curve). The incident collision temperature is 32 mK ($E^{*}=0.02228\;{\text{cm}}^{-1}$, horizontal gray dashed line), and rotational quantum numbers l=10−12. The black short lines indicate the quasibound states.

Distinct from quantum interference in nonresonant cases, the occurrence of quantum tunneling leads to a dominance of individual partial wave contributions. As shown in Fig. 8 (A and B), the ion energy distribution for PI at a collision temperature of 32 mK (incident collision energy $E^{*}=0.02228\;{\text{cm}}^{-1}$) is presented along with its partial-wave–resolved relative contributions. The analysis indicates that the *l* = 11 partial wave accounts for ∼64% of the integral cross section (ICS), representing the dominant contribution. Furthermore, Fig. 8C displays the angular DCS, featuring a strong forward signal and a secondary backward peak, the latter being a signature of the resonance.

**Fig. 9.**
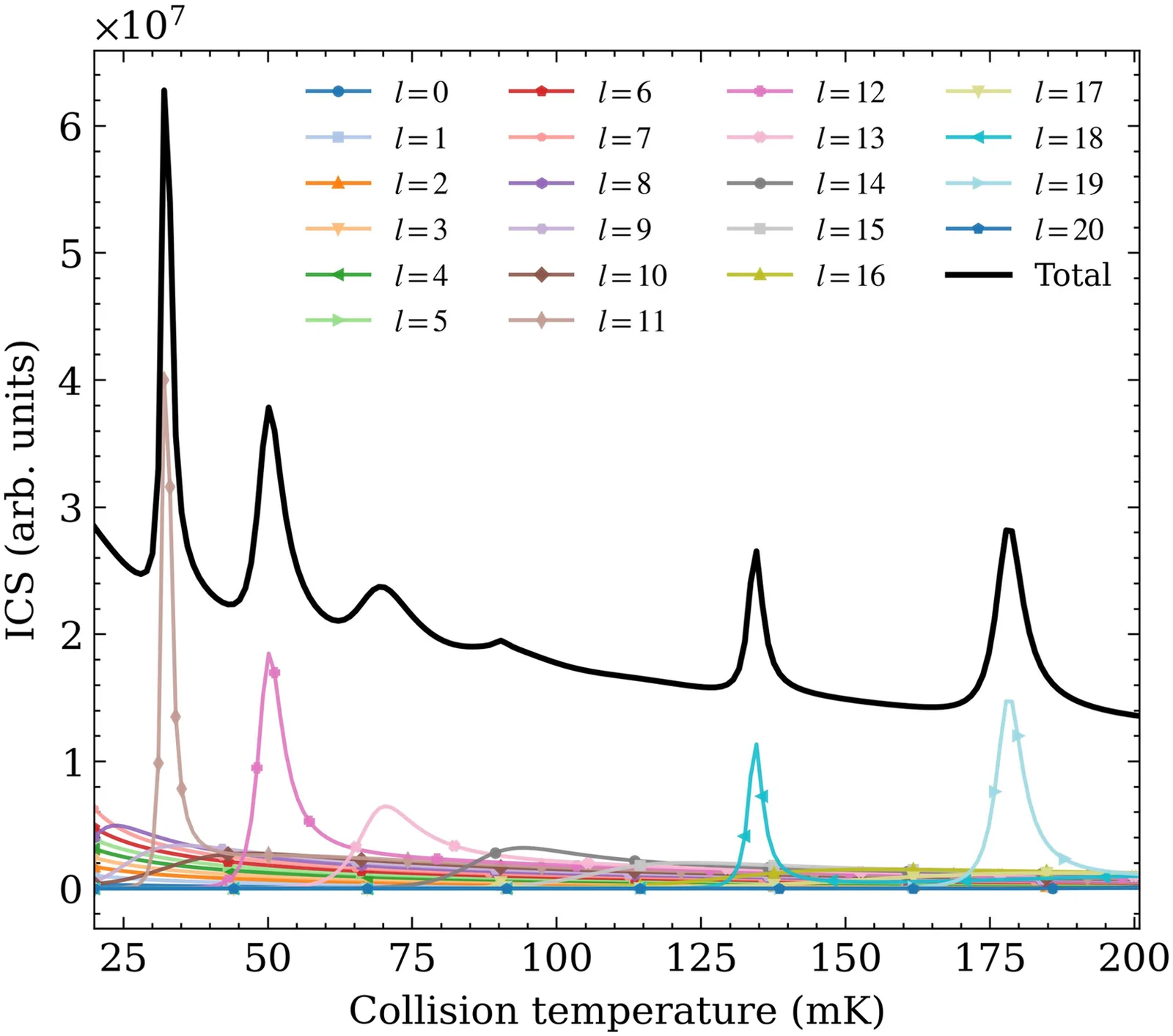
Contribution to the cross sections from different partial waves.

To elucidate the mechanism that results in this selectivity, Fig. 8 (D to F) compares the effective PECs and the quantity ${|\psi^{*}|}^{2}\times \Gamma$ for partial waves with *l* = 10, 11, and 12. Notably, the *l* = 11 partial wave exhibits an effective potential barrier height of $V_{b}=0.02775\;{\text{cm}}^{-1}$, which is slightly above the incident collision energy $E^{*}=0.02228\;{\text{cm}}^{-1}$. This configuration leads to an enhanced effective probability density ${|\psi^{*}|}^{2}\times \Gamma$ within the short-range potential well compared to the *l* = 10 and 12 cases. The pronounced tunneling penetration through the *l* = 11 barrier substantially amplifies the wave function density in the reaction region, thereby maximizing the PI probability. As for the nonresonant *l* = 10 and 12 cases, the centrifugal barrier for the *l* = 12 partial wave (Fig. 8F) lies well above the collision energy, so its contribution to the ICS is small, as the reaction is strongly suppressed. For the *l* = 10 partial wave, its centrifugal barrier is relatively low and does not exceed the collision energy (Fig. 8D). Consequently, its contribution to the ICS still exhibits a considerable magnitude (Fig. 9). Furthermore, for the Kr* + Rb collision system, the resonance peaks’ widths are substantially smaller than the energy spacings. Thus, when we examine the partial-wave scattering cross sections at 32 mK individually (Fig. 8B), only the *l* = 11 partial wave makes the dominant contribution due to its shape resonance. In contrast, the shape resonance peaks for the neighboring *l* = 10 and *l* = 12 partial waves are located far from 32 mK relative to their resonance widths, and thus their contributions to the scattering are negligible.

Considering the complex energy composition under experimental conditions, we propose that the observed enhancement of the backward-scattering distribution also incorporates contributions from resonances. The predicted collision energy dependence of partial wave DCS and ICS can further visualize the effect of resonances (Fig. 9). The *l*-dependent quasibound states trapped behind the centrifugal barriers exhibit similar energies, which nonetheless shift upward with increasing *l.* This trend is especially clear for the resonance series with *l* = 11, 12, and 13 in Fig. 9. As these resonances are well separated, even after summing over *l*, these resonant contributions are not completely washed out.

## References

[1] A. B. Henson, S. Gersten, Y. Shagam, J. Narevicius, E. Narevicius, Observation of resonances in penning ionization reactions at sub-kelvin temperatures in merged beams. Science 338, 234–238 (2012). 23066076 10.1126/science.1229141

[2] E. Lavert-Ofir, Y. Shagam, A. B. Henson, S. Gersten, J. Kłos, P. S. Żuchowski, J. Narevicius, E. Narevicius, Observation of the isotope effect in sub-kelvin reactions. Nat. Chem. 6, 332–335 (2014). 24651201 10.1038/nchem.1857

[3] A. Klein, Y. Shagam, W. Skomorowski, P. S. Żuchowski, M. Pawlak, L. M. C. Janssen, N. Moiseyev, S. Y. T. van de Meerakker, A. van der Avoird, C. P. Koch, E. Narevicius, Directly probing anisotropy in atom–molecule collisions through quantum scattering resonances. Nat. Phys. 13, 35–38 (2016).

[4] S. D. S. Gordon, J. J. Omiste, J. W. Zou, S. Tanteri, P. Brumer, A. Osterwalder, Quantum-state-controlled channel branching in cold Ne(^3^P_2_)+Ar chemi-ionization. Nat. Chem. 10, 1190–1195 (2018). 30297754 10.1038/s41557-018-0152-2

[5] J. Zou, S. D. S. Gordon, A. Osterwalder, Sub-kelvin stereodynamics of the Ne(^3^P_2_)+N_2_ reaction. Phys. Rev. Lett. 123, 133401 (2019). 31697548 10.1103/PhysRevLett.123.133401

[6] P. Paliwal, N. Deb, D. M. Reich, A. van der Avoird, C. P. Koch, E. Narevicius, Determining the nature of quantum resonances by probing elastic and reactive scattering in cold collisions. Nat. Chem. 13, 94–98 (2020). 33257885 10.1038/s41557-020-00578-x

[7] G. Q. Tang, M. Besemer, S. Kuijpers, G. C. Groenenboom, A. van der Avoird, T. Karman, S. Y. T. van de Meerakker, Quantum state–resolved molecular dipolar collisions over four decades of energy. Science 379, 1031–1036 (2023). 36893253 10.1126/science.adf9836

[8] S. E. J. Kuijpers, D. H. Parker, J. Loreau, A. van der Avoird, S. Y. T. van de Meerakker, Imaging scattering resonances in low-energy inelastic ND_3_-H_2_ collisions. Nat. Commun. 16, 7181 (2025). 40764320 10.1038/s41467-025-62511-5 PMC12325946

[9] A. T. Grier, M. Cetina, F. Oručević, V. Vuletić, Observation of cold collisions between trapped ions and trapped atoms. Phys. Rev. Lett. 102, 223201 (2009). 19658862 10.1103/PhysRevLett.102.223201

[10] M. Z. Walewski, M. D. Frye, O. Katz, M. Pinkas, R. Ozeri, M. Tomza, Quantum control of ion-atom collisions beyond the ultracold regime. Sci. Adv. 11, eadr8256 (2025). 39908382 10.1126/sciadv.adr8256 PMC11800771

[11] B. R. Heazlewood, T. P. Softley, Towards chemistry at absolute zero. Nat. Rev. Chem. 5, 125–140 (2021). 37117610 10.1038/s41570-020-00239-0

[12] L. D. Carr, D. DeMille, R. V. Krems, J. Ye, Cold and ultracold molecules: Science, technology and applications. New J. Phys. 11, 05504 (2009).

[13] V. Plomp, X. D. Wang, F. Lique, J. Kłos, J. Onvlee, S. Y. T. van de Meerakker, High-resolution imaging of C + He collisions using zeeman deceleration and vacuum-ultraviolet detection. J. Phys. Chem. Lett. 12, 12210–12217 (2021). 34928163 10.1021/acs.jpclett.1c03643 PMC8724800

[14] H. L. Bethlem, G. Berden, G. Meijer, Decelerating neutral dipolar molecules. Phys. Rev. Lett. 83, 1558–1561 (1999).

[15] S. Y. T. van de Meerakker, H. L. Bethlem, G. Meijer, Taming molecular beams. Nat. Phys. 4, 595–602 (2008).

[16] S. Y. T. van de Meerakker, H. L. Bethlem, N. Vanhaecke, G. Meijer, Manipulation and control of molecular beams. Chem. Rev. 112, 4828–4878 (2012). 22449067 10.1021/cr200349r

[17] A. von Zastrow, J. Onvlee, S. N. Vogels, G. C. Groenenboom, A. van der Avoird, S. Y. T. van de Meerakker, State-resolved diffraction oscillations imaged for inelastic collisions of NO radicals with He, Ne and Ar. Nat. Chem. 6, 216–221 (2014). 24557136 10.1038/nchem.1860

[18] J. Onvlee, S. D. S. Gordon, S. N. Vogels, T. Auth, T. Karman, B. Nichols, A. van der Avoird, G. C. Groenenboom, M. Brouard, S. Y. T. van de Meerakker, Imaging quantum stereodynamics through Fraunhofer scattering of NO radicals with rare-gas atoms. Nat. Chem. 9, 226–233 (2017). 28221351 10.1038/nchem.2640

[19] E. L. Raab, M. Prentiss, A. Cable, S. Chu, D. E. Pritchard, Trapping of neutral sodium atoms with radiation pressure. Phys. Rev. Lett. 59, 2631–2634 (1987). 10035608 10.1103/PhysRevLett.59.2631

[20] H. R. Thorsheim, J. Weiner, P. S. Julienne, Laser-induced photoassociation of ultracold sodium atoms. Phys. Rev. Lett. 58, 2420–2423 (1987). 10034744 10.1103/PhysRevLett.58.2420

[21] C. Regal, C. Ticknor, J. Bohn, D. S. Jin, Creation of ultracold molecules from a Fermi gas of atoms. Nature 424, 47–50 (2003). 12840753 10.1038/nature01738

[22] M. T. Hummon, M. Yeo, B. K. Stuhl, A. L. Collopy, Y. Xia, J. Ye, 2D magneto-optical trapping of diatomic molecules. Phys. Rev. Lett. 110, 143001 (2013). 25166984 10.1103/PhysRevLett.110.143001

[23] X. Y. Chen, S. Biswas, S. Eppelt, A. Schindewolf, F. Deng, T. Shi, S. Yi, T. A. Hilker, I. Bloch, X. Y. Luo, Ultracold field-linked tetratomic molecules. Nature 626, 283–287 (2024). 38297128 10.1038/s41586-023-06986-6 PMC10849947

[24] I. Kozyryev, L. Baum, K. Matsuda, B. L. Augenbraun, L. Anderegg, A. P. Sedlack, J. M. Doyle, Sisyphus laser cooling of a polyatomic molecule. Phys. Rev. Lett. 118, 173201 (2017). 28498706 10.1103/PhysRevLett.118.173201

[25] E. S. Shuman, J. F. Barry, D. DeMille, Laser cooling of a diatomic molecule. Nature 467, 820–823 (2010). 20852614 10.1038/nature09443

[26] H. Yang, X. Y. Wang, Z. Su, J. Cao, D. C. Zhang, J. Rui, B. Zhao, C. L. Bai, J. W. Pan, Evidence for the association of triatomic molecules in ultracold ^23^Na^40^K + ^40^K mixtures. Nature 602, 229–233 (2022). 35140383 10.1038/s41586-021-04297-2

[27] F. Fang, W. C. Zhou, Y. F. Li, D. B. Qian, C. J. Luo, D. M. Zhao, X. W. Ma, J. Yang, Design and characterization of a velocity-map imaging apparatus for low-energy photo-ion spectroscopy using magneto-optically trapped atoms. Rev. Sci. Instrum. 92, 043103 (2021). 34243435 10.1063/5.0033595

[28] J. J. Park, Y. K. Lu, A. O. Jamison, T. V. Tscherbul, W. Ketterle, A Feshbach resonance in collisions between triplet ground-state molecules. Nature 614, 54–58 (2023). 36725997 10.1038/s41586-022-05635-8

[29] Y. Liu, K. K. Ni, Bimolecular chemistry in the ultracold regime. Annu. Rev. Phys. Chem. 73, 73–96 (2022). 34890257 10.1146/annurev-physchem-090419-043244

[30] Y. Liu, M. G. Hu, M. A. Nichols, D. Z. Yang, D. Q. Xie, H. Guo, K. K. Ni, Precision test of statistical dynamics with state-to-state ultracold chemistry. Nature 593, 379–384 (2021). 34012086 10.1038/s41586-021-03459-6

[31] J. Cao, B. Y. Wang, H. Yang, Z. J. Fan, Z. Su, J. Rui, B. Zhao, J. W. Pan, Observation of photoassociation resonances in ultracold atom-molecule collisions. Phys. Rev. Lett. 132, 093403 (2024). 38489622 10.1103/PhysRevLett.132.093403

[32] M. Chilcott, J. F. E. Croft, R. Thomas, N. Kjærgaard, Microscopy of an ultranarrow Feshbach resonance using a laser-based atom collider: A quantum defect theory analysis. Phys. Rev. A 106, 023303 (2022).

[33] S. Falcinelli, F. Pirani, F. Vecchiocattivi, The possible role of penning ionization processes in planetary atmospheres. Atmosphere 6, 299–317 (2015).

[34] A. T. J. B. Eppink, D. H. Parker, Velocity map imaging of ions and electrons using electrostatic lenses: Application in photoelectron and photofragment ion imaging of molecular oxygen. Rev. Sci. Instrum. 68, 3477–3484 (1997).

[35] S. Y. Liu, Y. C. Wang, R. F. Wu, G. M. Yang, W. Jiang, A Kr^*^–Rb cold collision apparatus based on atom trap. Rev. Sci. Instrum. 94, 043303 (2023). 38081251 10.1063/5.0137853

[36] M. Kasevich, D. S. Weiss, E. Riis, K. Moler, S. Kasapi, S. Chu, Atomic velocity selection using stimulated Raman transitions. Phys. Rev. Lett. 66, 2297–2300 (1991). 10043449 10.1103/PhysRevLett.66.2297

[37] R. Legere, K. Gibble, Quantum scattering in a juggling atomic fountain. Phys. Rev. Lett. 81, 5780–5783 (1998).

[38] P. E. Siska, Molecular-beam studies of Penning ionization. Rev. Mod. Phys. 65, 337–412 (1993).

[39] F. M. Penning, Über ionisation durch metastabile atome. Naturwissenschaften 15, 818 (1927).

[40] H. Hotop, T. E. Roth, M. W. Ruf, A. J. Yencha, Diatomic potential well depths from analyses of high-resolution electron energy spectra for autoionizing collision complexes. Theor. Chem. Acc. 100, 36–50 (1998).

[41] B. Ohayon, H. Rahangdale, J. Chocron, Y. Mishnayot, R. Kosloff, O. Heber, G. Ron, Imaging recoil ions from optical collisions between ultracold, metastable neon isotopes. Phys. Rev. Lett. 123, 063401 (2019). 31491183 10.1103/PhysRevLett.123.063401

[42] H. J. Werner, P. J. Knowles, G. Knizia, F. R. Manby, M. Schütz, Molpro: A general-purpose quantum chemistry program package. WIREs Comput. Mol. Sci. 2, 242–253 (2012).

[43] R. J. Le Roy, A. Pashov, betaFIT: A computer program to fit pointwise potentials to selected analytic functions. J. Quant. Spectrosc. Radiat. Transf. 186, 210–220 (2017).

[44] D. Bellert, W. H. Breckenridge, Bonding in ground-state and excited-state A^+^·Rg van der Waals ions (A= atom, Rg= rare-gas atom): A model-potential analysis. Chem. Rev. 102, 1595–1622 (2002). 11996545 10.1021/cr980090e

[45] A. D. Koutselos, E. A. Mason, L. A. Viehland, Interaction universality and scaling laws for interaction potentials between closed-shell atoms and ions. J. Chem. Phys. 93, 7125–7136 (1990).

[46] W. H. Breckenridge, V. L. Ayles, T. G. Wright, Analysis of the bonding in alkali-cation/Rg complexes (Rg= He–Xe) using a simple model potential. Chem. Phys. 333, 77–84 (2007).

[47] L. Thiel, H. Hotop, W. Meyer, Ab initio investigation of the autoionization process Ar* (4s ^3^P_2_, ^3^P_0_)+Hg→(Ar–Hg)^+^+e^−^: Potential energy curves and autoionization widths, ionization cross sections, and electron energy spectra. J. Chem. Phys. 122, 184309 (2005). 15918706 10.1063/1.1891666

[48] D. Z. Yang, H. Guo, A time-dependent quantum approach to dissociative recombination, associative ionization, and penning ionization. J. Chem. Phys. 159, 044105 (2023). 37486044 10.1063/5.0156998

[49] R. J. Bieniek, Semi-classical uniform approximation in penning ionization. J. Phys. B At. Mol. Phys. 7, L266–L270 (1974).

[50] A. Khan, H. R. Siddiqui, P. E. Siska, Angle-energy distributions of Penning ions in crossed molecular beams. II. Effect of Penning electron recoil in Ne*(3*s* ^3^P_2_)+H, D→Ne+H^+^, D^+^+e^−^. J. Chem. Phys. 95, 3371–3380 (1991).

[51] V. Dribinski, A. Ossadtchi, V. A. Mandelshtam, H. Reisler, Reconstruction of Abel-transformable images: The Gaussian basis-set expansion Abel transform method. Rev. Sci. Instrum. 73, 2634–2642 (2002).

[52] P. Langevin, Une formule fondamentale de théorie cinétique. Ann. Chim. Phys. 5, 245–288 (1905).

[53] A. Blech, Y. Shagam, N. Hölsch, P. Paliwal, W. Skomorowski, J. W. Rosenberg, N. Bibelnik, O. Heber, D. M. Reich, E. Narevicius, C. P. Koch, Phase protection of Fano-Feshbach resonances. Nat. Commun. 11, 999 (2020). 32081896 10.1038/s41467-020-14797-w PMC7035365

[54] F. Schreck, K. van Druten, Laser cooling for quantum gases. Nat. Phys. 17, 1296–1304 (2021).

[55] N. J. Fitch, M. R. Tarbutt, Chapter three—Laser-cooled molecules. Adv. At. Mol. Opt. Phys. 70, 157–262 (2021).

[56] P. Fröbrich, I. I. Gontchar, Langevin description of fusion, deep-inelastic collisions and heavy-ion-induced fission. Phys. Rep. 292, 131–237 (1998).

[57] C. M. Bender, S. A. Orszag, “Asymptotic expansion of integrals,” in *Advanced Mathematical Methods for Scientists and Engineers I* (Springer, 1999), pp. 277–278.

[58] R. J. Bieniek, Complex potential and electron spectrum in atomic collisions involving fast electronic transitions: Penning and associative ionization. Phys. Rev. A 18, 392–413 (1978).

[59] B. R. Johnson, New numerical methods applied to solving the one-dimensional eigenvalue problem. J. Chem. Phys. 67, 4086–4093 (1977).

