# Supplementary material for: Direct observation of quantum scattering oscillations in the controlled Kr* + Rb cold collision
Source: Sci Adv. 2026 Sep 4;12(36):eaeh1789. doi: 10.1126/sciadv.aeh1789 (PMC13544253; doi:10.1126/sciadv.aeh1789)
Supplement: Supplementary file 1 [file sciadv.aeh1789_sm.pdf]

Science Advances
AAAS

Supplementary Materials for

Direct observation of quantum scattering oscillations in the controlled Kr* + Rb cold collision

Yu-Chan Wang *et al.*

Corresponding author: Ya-Fu Guan, guanyf@dicp.ac.cn; Bin Zhao, zhaobin@sustech.edu.cn; Wei Jiang, wjiang1@ustc.edu.cn

Sci. Adv. **12**, eaeh1789 (2026)
DOI: 10.1126/sciadv.aeh1789

This PDF file includes:

Supplementary Text
Figs. S1 to S14
Tables S1 and S2

Supplementary Text

1. Experimental Time Sequence

The time sequence is shown as Fig S1. Following the loading of the Kr* MOT (designated as time zero), the MOT's quadrupole magnetic field ($B_{Kr^*\ MOT}$) and Zeeman Slower (ZS) light are switched off to form an optical molasses. After 3.5 ms, based on the predetermined launch velocity (collision temperature), the frequencies of the two vertical MOT beams (Kr* MM^+ Freq and Kr* MM^- Freq) are ramped up and down respectively in 1.5 ms, creating a Moving Molasses to launch the atoms. Subsequently, the detuning of all MOT beams (Kr* $MM^\pm$ Freq, Kr* MOT H Freq) is rapidly increased and their intensity (Kr* MOT Amp) is reduced to apply polarization gradient cooling to the launched Kr* atomic cloud, reducing its temperature (energy width) in its own moving frame to approximately 20 μK. To ensure all Rb atoms are in the ground state during Kr* + Rb collisions, the Rb MOT light is completely switched off 1 ms before the Kr* cloud reaches the collision region, allowing the Rb atoms to decay to the ground state ($5S_{1/2}$, F=2). Subsequently, the Kr* and Rb clouds collide, while the CMOS camera behind the MCP phosphor screen is triggered to collect the VMI signal of the resultant ions.

2. Background Processing of VMI Images

The VMI ion signal detected on the MCP comprises not only ions produced from Kr* + Rb collisions but also a small Kr^+ ion background generated via Kr* + Kr* collisions within the launched Kr* cloud. The Kr^+ ion background count rate ranges from 2 to 15 counts/frame, accounting for 10%-20% of the count rate of the Kr* + Rb collision ion products. Background signals from other sources, including photoionization of Rb atoms in the MOT by ambient light, collisional ionization between Kr* and uncooled background thermal Rb atoms, and the dark counts of the MCP, are negligible with a count rate ~1 count/frame in total.

To obtain the distribution of the Kr^+ background in the VMI image, a background image B (Fig S2(a)) was acquired by accumulating $>10^5$ background ion counts in the absence of the Rb MOT. As the intensity of the background signal during experiments does not perfectly match the pre-acquired background intensity, a global scaling factor is applied to image B for correction. The correction procedure is as follows: a region S_n without the Kr* + Rb collision ion signals is selected in the VMI image S, and an identical region B_n is selected in the background image B. The ratio r of the total ion counts within region Sn to those in Bn serves as the correction factor. The intensity of B is then adjusted accordingly, yielding the background image B' = *r* B. Finally, to reduce the noise due to the finite counts, the background image B' undergoes Gaussian smoothing (σ = 4.2 px) to produce B_s (Fig S2(b)). This smoothed background image Bs is subtracted from VMI image S to obtain the background-free VMI image S' = S - B_s.

3. Abel Inversion Algorithm

The program based on the BASEX algorithm is utilized to perform the Abel inversion on the VMI images. To mitigate noise on the symmetry axis and reduce the impact of dead pixels from the CMOS camera, the array S' was subjected to Gaussian smoothing prior to inversion. Since the inversion algorithm operates on a row-by-row basis, suppressing noise on the symmetry axis primarily requires enhanced filtering along the x-direction; consequently, an anisotropic Gaussian kernel (σ_x = 5.7, σ_z = 1.4) was employed, which yielded high-quality images (Fig. 2 A, E ,I , Fig S4 and Fig S5). However, this preprocessing step introduces noticeable distortion to the signal from low velocity associative ionization products located at the center of the VMI image. It also blurs details in the ion kinetic energy spectrum and broadens the forward peak in the angular

differential cross section (DCS). Omitting this denoising step preserves more details in the DCS at the expense of more measurement and fitting noise (Fig S3, b). Radial and angular integration of the inverted image yields the kinetic energy spectrum and the angular differential cross section (DCS) of the ion products, respectively (Fig 2 C, D, G, H, K, L, and Fig S3, C and D). To achieve a better trade-off between noise suppression and detail preservation in both the kinetic energy spectrum and the angular DCS, an isotropic Gaussian kernel ($\sigma_x = \sigma_z = 1.4$) was applied (Fig 2, C, D, G, H, K and L) in comparison with the theoretical calculations. Furthermore, to prevent interference from the distorted low-velocity ion products on the angular DCS, only ion products with velocities greater than 150 m/s were used in the calculation. To compare with theoretical calculation, the same procedure was performed on simulated VMI images (Fig 2 B, C, D, F, G, H, J, K and L).

4. *Ab initio* treatment of Kr* + Rb

In treating the electronic states of Kr*Rb penning ionization system, a principal difficulty arises due to the fact that Kr* + Rb states are submerged in the continuum of states of the $(KrRb)^+$ + e^- products. The electronic wave function of the latter cannot be correctly described by standard electronic structure methods designed for the description of bound states and the variational principle is expected to drive Kr* + Rb states down either to the ground state or one of the excited bound states. Therefore, during the wave function optimization, one should seek to impose a constraint on the electron configurations to maintain consistency with Kr* + Rb states. To this end, the restricted active space self-consistent-field (RASSCF) method was adopted in this work.

Two atoms are placed on the z axis. At first, a restricted open-shell Hartree-Fock (ROHF) calculation for the ground state KrRb was performed, with two well separated atoms (100 a_0). The molecular orbitals are then essentially the atomic orbitals, from which the $4p_x$, $4p_y$, $4p_z$ and 5s orbitals of Kr and the 5s orbital of Rb can be identified. In the following RASSCF calculation, the 5s orbitals of both Rb and Kr were kept singly occupied and one of the 4p orbitals of Kr was also singly occupied.

If the singly occupied 4p orbital of Kr is $4p_z$, then the electronic state is denoted as Σ. If the singly occupied 4p orbital of Kr is $4p_x$ ($4p_y$), then the electronic state is denoted as Π_x (Π_y). Π_x and Π_y are degenerate due to symmetry. 1,2 $^2\Sigma$, 1,2 $^2\Pi_x$, 1,2 $^2\Pi_y$, $^4\Sigma$, $^4\Pi_x$ and $^4\Pi_y$were averaged in the RASSCF calculation. The basis set for Kr is aug-cc-pCVQZ, and the basis set for Rb is aug-cc-pwCVQZ-PP. All electronic structure data were computed with Molpro program (*42*).

To avoid discontinuity of PEC due to the orbital changes in RASSCF, the orbitals from the Kr* + Rb system at large internuclear distances were used as the initial guesses for subsequent RASSCF calculations conducted at smaller internuclear distances. The CASSCF (complete active space self-consistent field) calculations for the ground state (Kr $4p^6$ Rb $5s^1$) were also performed. The energy difference between RASSCF and CASSCF remains approximately 80 000 cm^{-1}, which corresponds to the excitation energy of Kr atom (4p→5s). This further confirms that the RASSCF calculations have converged to the right configuration (Kr $4p^5 5s^1$ Rb $5s^1$).

As can be seen from Fig S6, three groups of PECs with similar shape can be observed: (1) $^4\Sigma$, $^4\Pi_x$ and $^4\Pi_y$, (2) $1^2\Sigma$, $1^2\Pi_x$ and $1^2\Pi_y$, (3) $2^2\Sigma$, $2^2\Pi_x$ and $2^2\Pi_y$. Within each group, the potential energy curves for the Σ and Π states are very close to each other, indicating that the interaction potential is mainly determined by the interaction between the Kr 5s electron and the Rb 5s electron, while the Kr ionic core acts as a perturbation.

When spin-orbit coupling is considered, the total electronic Hamiltonian H^e, comprises a spin-free component (H_{SF}) and a spin-orbit coupling component (H_{SO}):

$$H^e = \mathrm{H}_{SF} + \mathrm{H}_{SO} \tag{S1}$$

The electronic states depicted in Fig S6 are the eigenstates of H_{SF}. Once the wavefunctions of the spin-free eigenstates are determined, the spin-orbit coupling elements between them can be readily evaluated. By diagonalizing the total Hamiltonian matrix (H^e), the spin-free eigenstates will split due to spin-orbit couplings.

Next, we give an example of the calculation. The internuclear distance is set to R = 20 a_0. The total Hamiltonian is written as follows:

$$H^e(t) = \mathrm{H}_{SF} + t \cdot \mathrm{H}_{SO} \tag{S2}$$

where $0 \leq t \leq 1$. As t varies from 0 to 1, H_{SO} is gradually added to H_{SF}. By diagonalizing $H^e(t)$ at each value of t, the details of electronic state splitting can be obtained (see Fig S7, S8 and S9).

As can be seen from Fig S10, when Kr* and Rb are widely separated, the PECs can be classified into four groups with degeneracies of 5, 3, 1, and 3, which is consistent with the splitting of Kr* energy levels. The relative energies of degenerate groups are 0.00, 1267.17, 4774.79, and 5827.09 cm^{-1}, respectively, which are in good agreement with the experimentally determined splittings (0.00, 945.026, 5219.87, and 5874.96 cm^{-1}), thereby verifying the validity of RASSCF wavefunctions.

5. Analytical function form for Kr* + Rb PEC

In this work, the Kr* + Rb collision is assumed to occur on the lowest PEC in Fig S10, i.e. one of the $|\Omega| = 1/2$ channels, which correlates adiabatically to the Kr* 5s [$^2P_{3/2}$] (J=2) + Rb ^2S atomic terms.

To analytically express this PEC, the Morse/Long-Range (MLR) potential function was adopted[42], which takes the following form

$$V_{MLR}(r) = D_e \left\{1 - \frac{u_{LR}(r)}{u_{LR}(r_e)} e^{-\beta(r)\cdot y_p^{r_e}(r)}\right\}^2 \tag{S3}$$

where D_e is the well depth, r_e the equilibrium internuclear distance, the exponent coefficient $\beta(r) = \beta_{MLR}(y_{p/q}^{ref}(r))$ is a relatively slowly varying function of r, and $u_{LR}(r)$ incorporates the attractive limiting long-range form, given by:

$$u_{LR}(r) = \frac{C_{m_1}}{r^{m_1}} + \frac{C_{m_2}}{r^{m_2}} + \cdots + \frac{C_{m_{last}}}{r^{m_{last}}} \tag{S4}$$

and $u_{LR}(r_e)$ denotes its value at r_e. In this work, only the C_6 term is included. The Levenberg-Marquardt algorithm was used to fit the *ab initio* energy data.

To demonstrate the sensitivity of cold collision reactions to long-range interactions, two different C_6 coefficients were used in the subsequent simulations: one derived from a fit to the *ab initio* calculation, and the other obtained by fitting the period of the experimentally measured angular oscillation using the Langevin model (See Section 9).

The imaginary part of the PEC was modeled using an exponential function,

$$\Gamma = A \exp\left(-\frac{r}{r_0}\right) \tag{S5}$$

with parameters $A = 5\times10^{-7}$ hartree and $r_0 = 10.0$ a_0 chosen to match the experimental data.

6. Kr + Rb^+ PEC

The electronic structure of Kr + Rb^+ is relatively simple, and extensive work has been done on the interaction between alkali-metal cations (A^+) and a rare gas partner (Rg). In this work, the following potential function is chosen to model the Kr + Rb^+ PEC,

$$V(R) = -\frac{\alpha_{RgD}Z^2}{2R^4} - \frac{C_6}{R^6} - \frac{\alpha_{RgQ}Z^2}{2R^6} + \frac{B_{Rg}Z^3}{2R^7} - \frac{C_8}{R^8} - \frac{\alpha_{RgO}Z^2}{2R^8} - \frac{\gamma Z^4}{24R^8} + Ae^{-bR} \tag{S6}$$

where Z is the "effective" charge on the A^+ ion; α_{RgD}, α_{RgQ}, and α_{RgO} are the dipole, quadrupole, and octupole polarizabilities of the Rg atom, respectively; B_{Rg} is the higher-order "dipole-quadrupole" polarizability of the Rg atom; γ is the higher-order "second dipole hyperpolarizability" of the Rg atom; and C_6 and C_8 are Z-independent coefficients representing the first and second terms in the dispersion interaction.

The parameters that define the Kr + Rb^+ PEC are listed in Table S2.

7. Ion energy distribution

To account for the experimental energy spreading, an energy-dependent convolution with a variance of $\sigma_{E^+} = 0.08\sqrt{E^+}$ is applied to the raw numerical ion energy distribution results. The computed results are shown in Fig S11, where the initial collision temperature is 30 mK.

As illustrated in Fig S11, the ion energy distribution magnitude is significant at lower E^+, peaks at $E^+ = 0.153$ eV, and then rapidly diminishes to zero. This theoretical result qualitatively agrees with experimental observations.

8. DCS Computation

To elucidate the experimentally observed angular interference pattern, we computed the theoretical angular distribution of ions produced through Penning ionization. Figure S12 presents the angular distributions of Rb^+ at different collision temperatures: a) 40 mK, b) 200 mK, and c) 5100 mK. The theoretical results consistently exhibit oscillatory structures in the angular distributions, which are characteristic of quantum interference between partial waves in the scattering process. Furthermore, the oscillation frequency increases with rising collision temperature. (The number of peaks between 0° and 180° is related to the maximum partial wave l_{max}, which, as discussed earlier, depends on the collision energy.)

Figure S13 presents the angular-resolved ion velocity distributions for partial waves $l = 9$–12 at an initial collision temperature of 40 mK. Panel (a) shows the relative contributions of these partial waves to the total DCS, normalized to 100%. As shown in panels (b)–(e), each individual l-partial wave exhibits symmetric forward–backward scattering in its velocity distribution. In contrast, the total ion velocity distribution in panel (f), which has contributions from all partial waves only displays a strong forward-scattering due to interference, i.e. the destructive interference strongly suppresses the backward scattering.

9. Modification of Kr* + Rb PEC

Differential cross sections (DCS) were computed using three distinct potential energy curves: an *ab initio* curve and two empirically modified curves. The empirical modifications involved not only adjusting the long-range C_6 coefficient but also adopting different functional forms to smoothly join the long-range and short-range regions of the potential. The *ab initio* potential qualitatively reproduces the experimentally measured angular distribution. However, the period of its angular oscillations shows significant deviation from the −1/3 power law, as illustrated in the upper panel of Fig. 4**B**. In contrast, the potentials modified using experimental results and Langevin theory more accurately reproduce the oscillation period of the angular distribution, as shown in the middle and lower panels of Fig. 4**B**. Nonetheless, finer details of the angular distribution remain sensitive to the intermediate-range part of the potential—particularly the collision energies at which backscattering enhancement occurs, which correspond to resonance energies (Fig. 4**B** middle and lower panels). This underscores the high sensitivity of both the DCS and resonance phenomena in low-energy collisions to the mid- to long-range potential. Thus, these experimental results provide a stringent benchmark for future theoretical calculations aiming for higher precision.

10. Phase Shift analysis

We selected collision energies of 160 mK and 177 mK as examples to illustrate the complex phase η_l of the S-matrix elements, $\langle\psi^+|\sqrt{\Gamma}|\psi^*\rangle$, which roughly corresponds to the sum of the phase shifts of ψ^+ and ψ^*. Analysis shows that this phase varies smoothly for the partial waves with $l < l_{max} \approx 18$. Note that η_l varies more slowly in the vicinity of l_{max}.

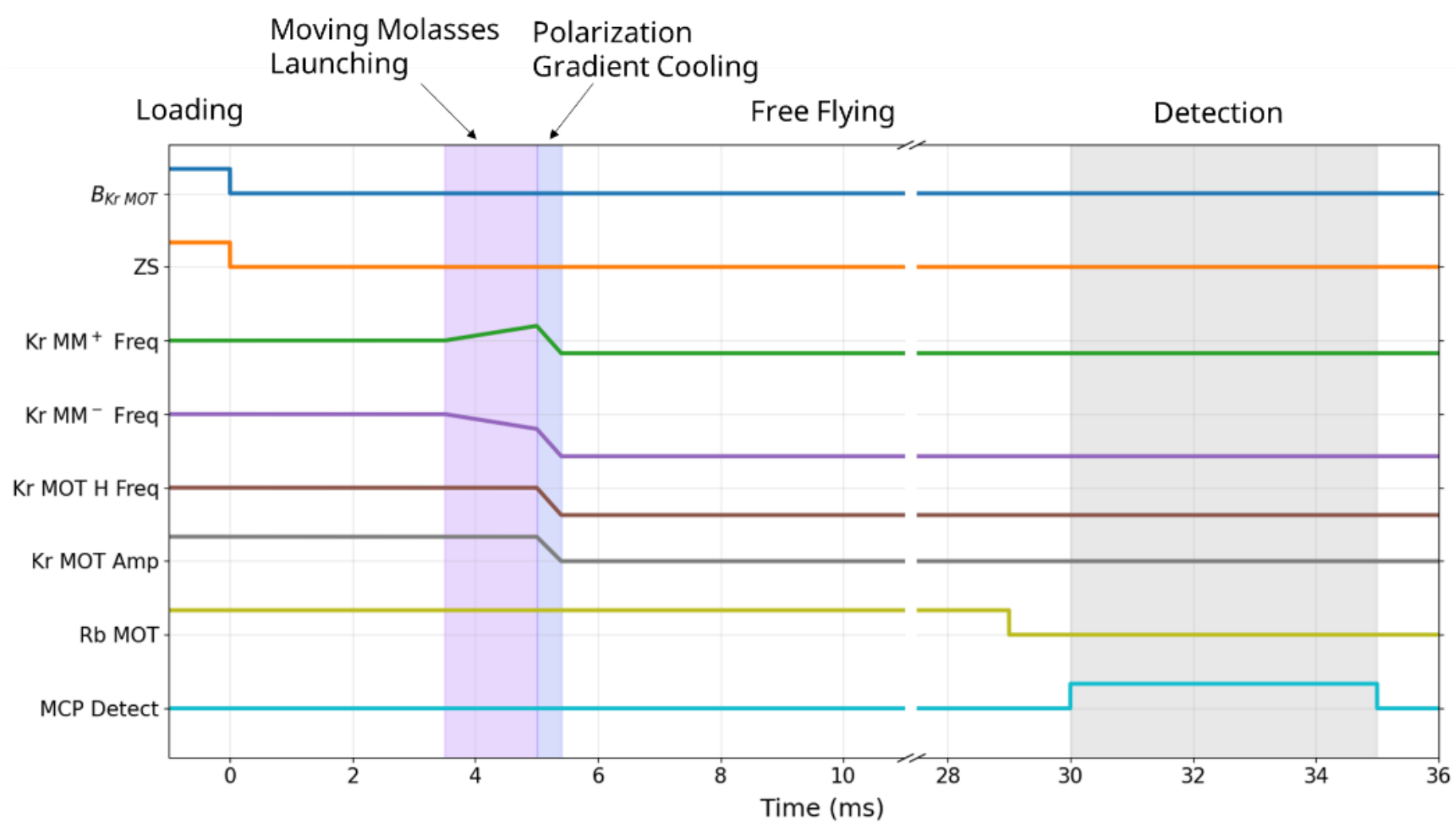

Fig. S1. The time sequence of the experiment.

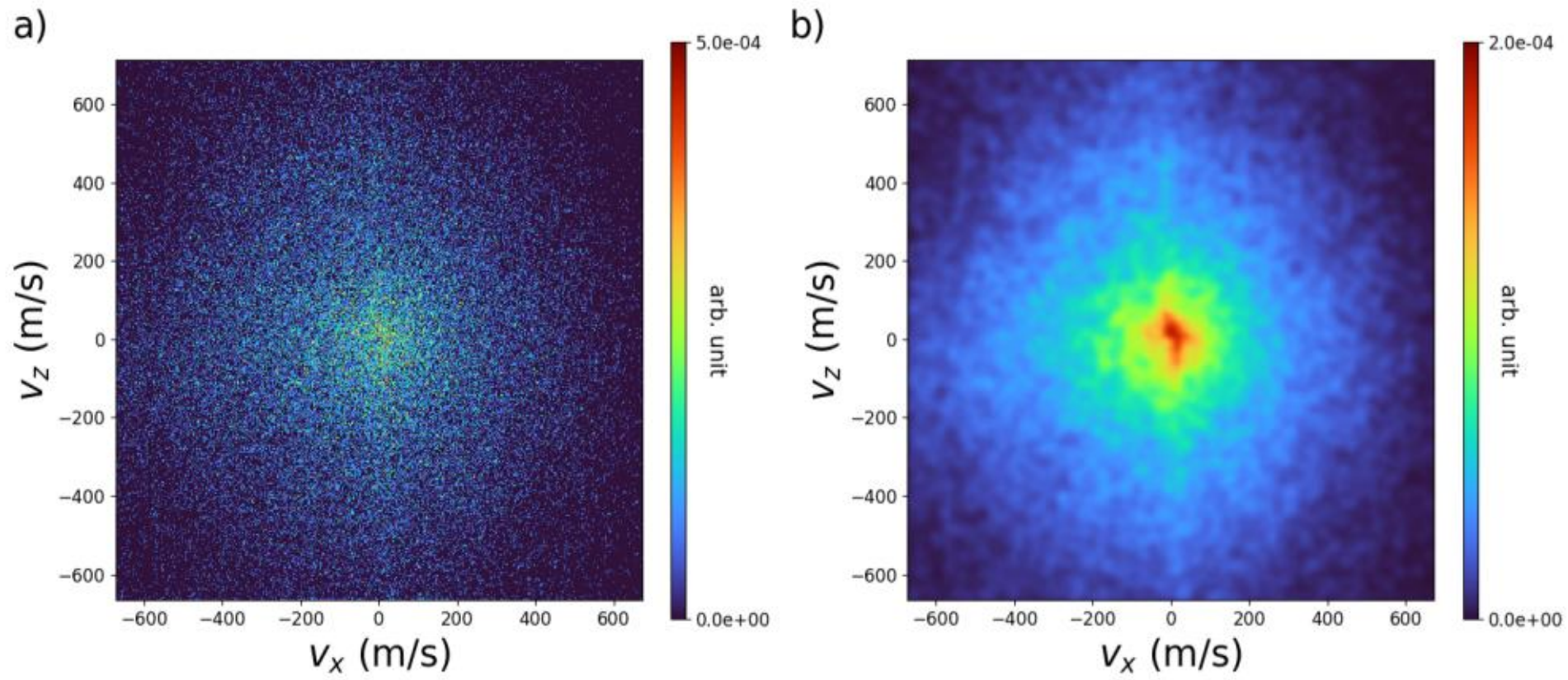

Fig S2. Background Processing of VMI Images. (a) Background signal of the VMI image measured at a collision energy of 120 mK. (b) The VMI image background signal after Gaussian smoothing.

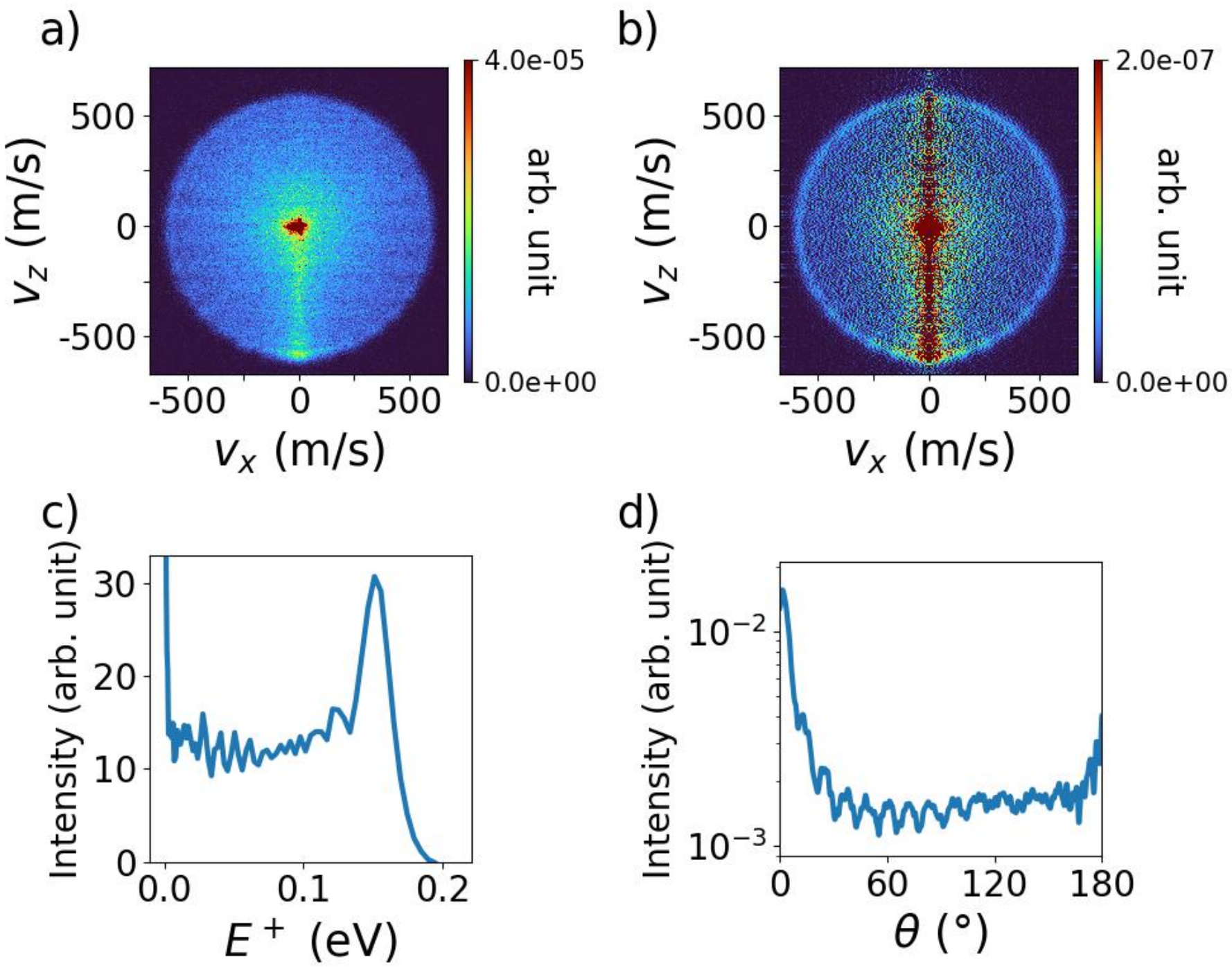

Fig S3. Results derived from the Abel inversion of the unsmoothed VMI image. (a) Experimentally acquired VMI image (unsmoothed, background-subtracted). (b) DCS obtained from Abel inversion. (c) Ion kinetic energy distribution. (d) Angular DCS. The collision energy is 120 mK.

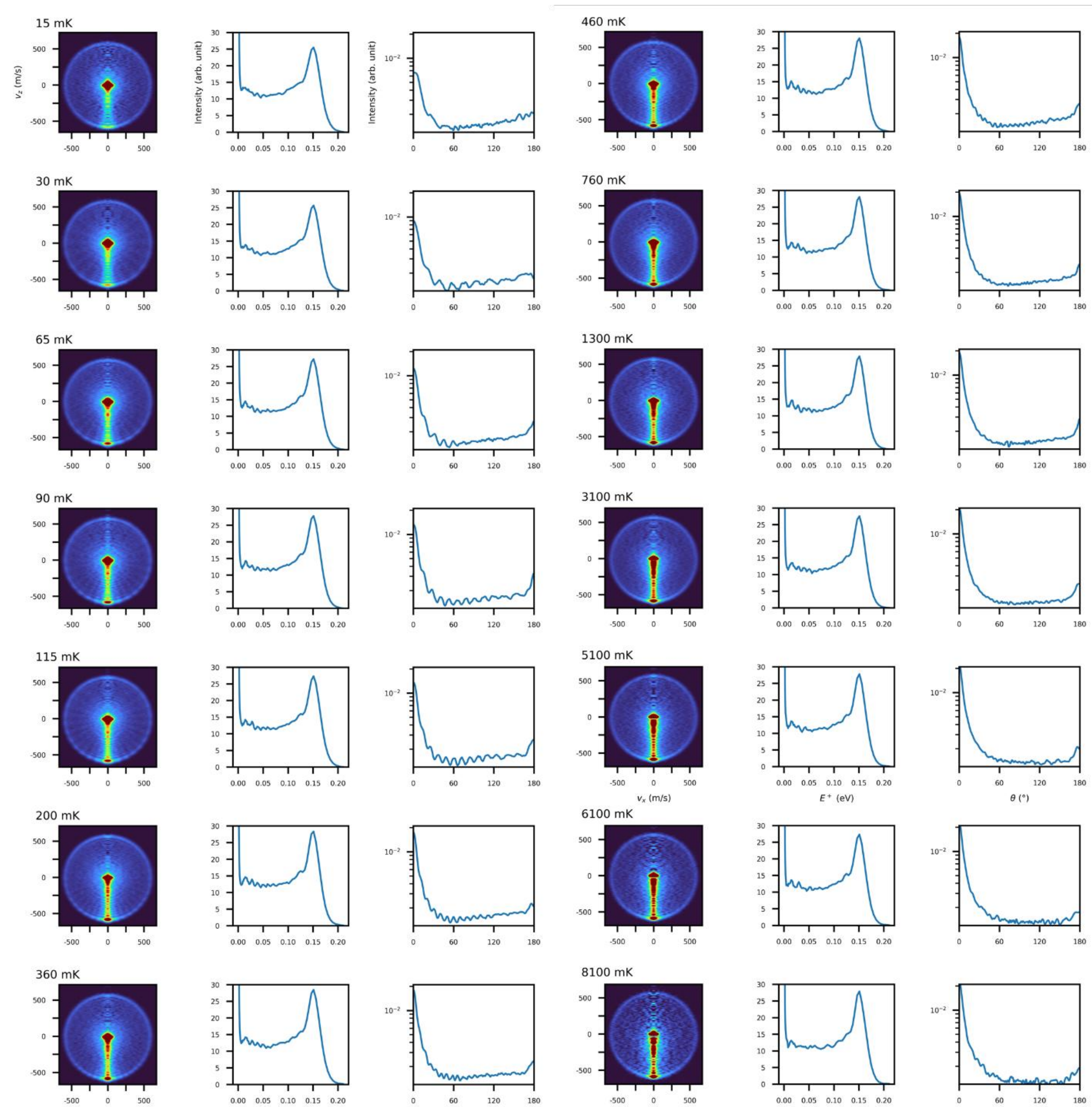

Fig S4. Several DCS measurements obtained at collision energies ranging from 15 mK to 8100 mK. The corresponding VMI images were processed with a Gaussian smoothing filter.

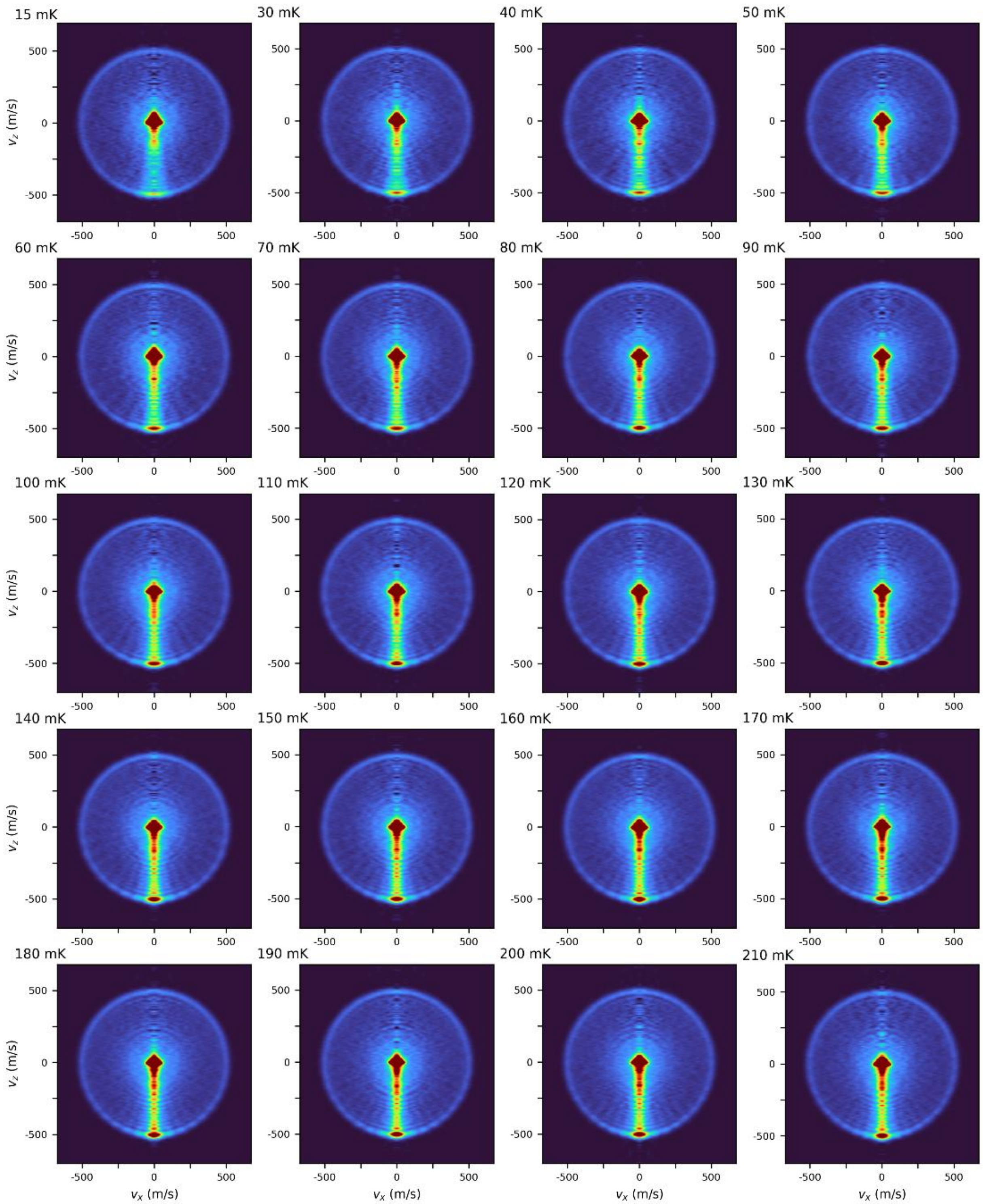

Fig S5. DCS measurements obtained at collision energies ranging from 15 mK to 210 mK. These are the basis of Fig. 3a. The corresponding VMI images were processed with a Gaussian smoothing filter.

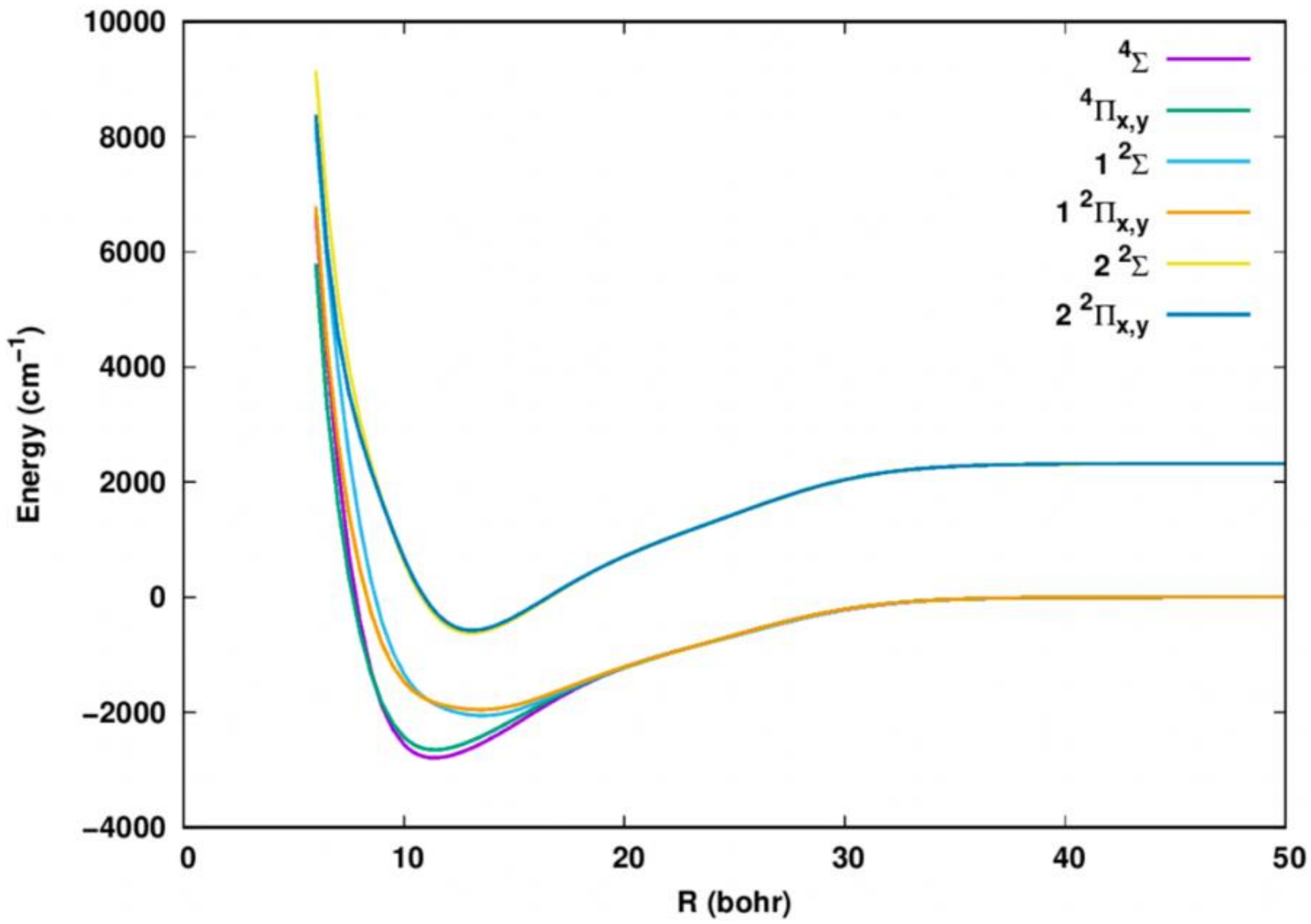

Fig S6. Potential energy curves of Kr* + Rb without spin-orbit splitting.

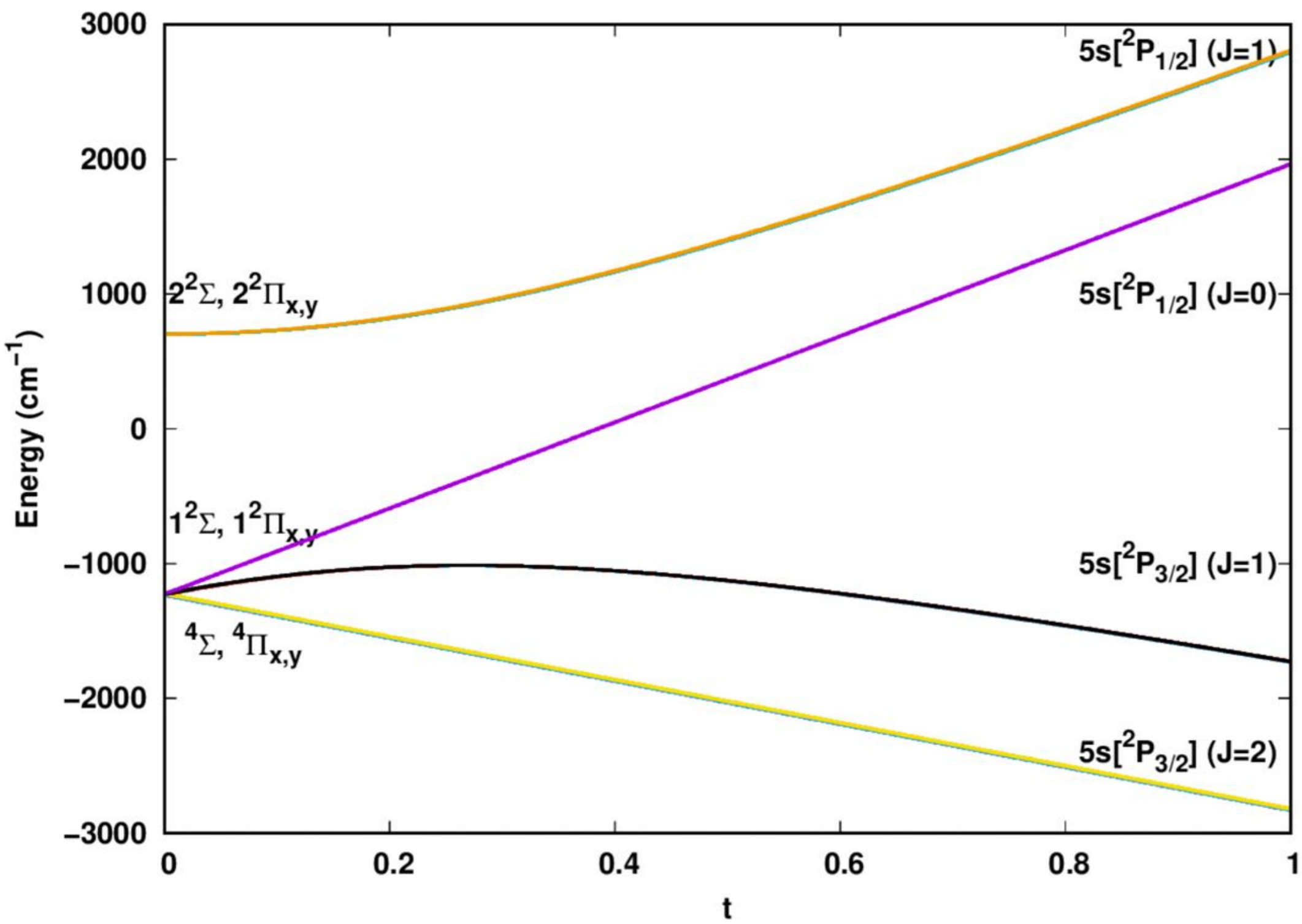

Fig S7. The spin-orbit splitting of $^4\Sigma$, $^4\Pi_{x,y}$, $1^2\Sigma$, $1^2\Pi_{x,y}$, $2^2\Sigma$ and $2^2\Pi_{x,y}$ at R = 20 a_0.

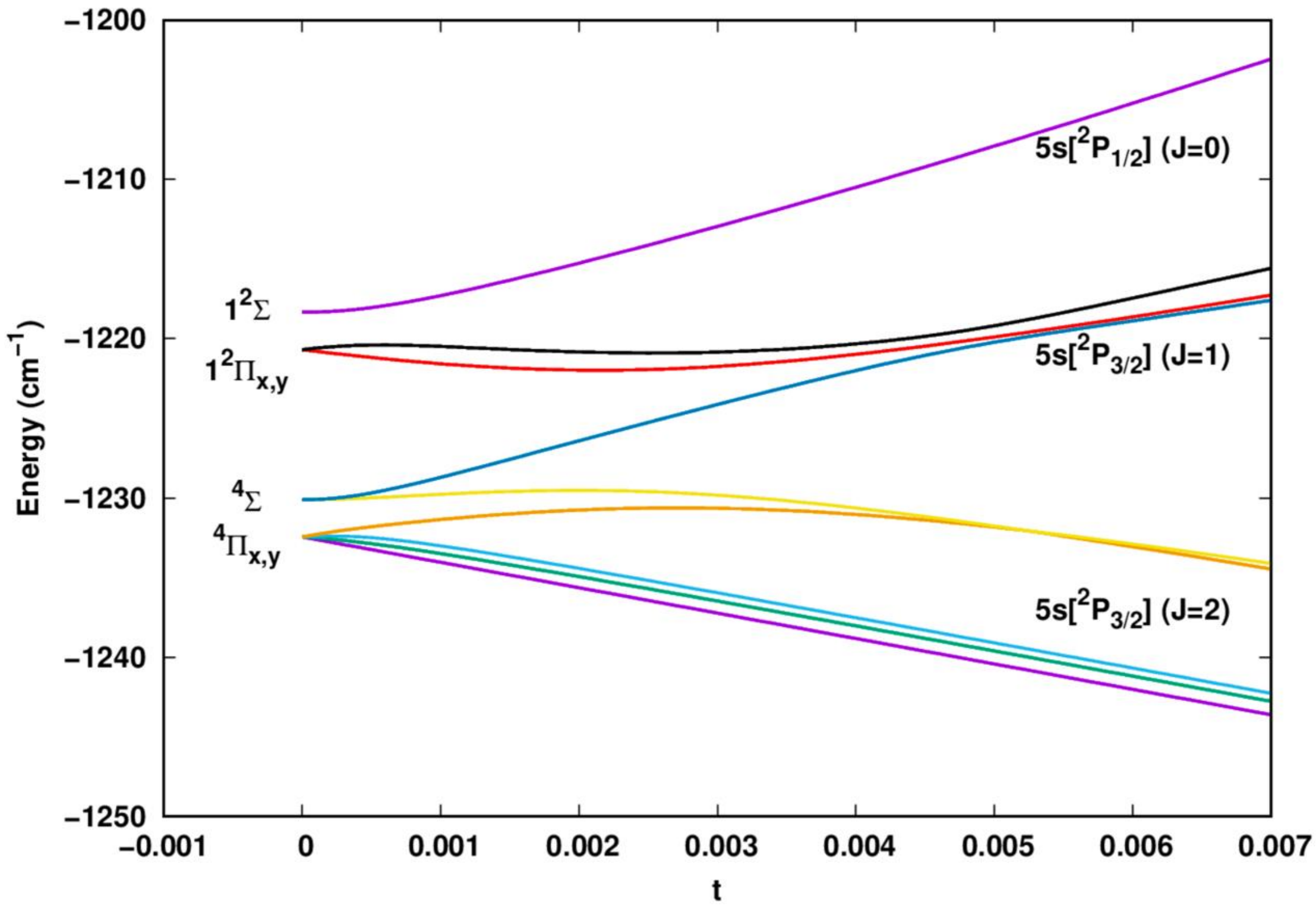

Fig S8. The spin-orbit splitting of $^4\Sigma$, $^4\Pi_{x,y}$, $1^2\Sigma$ and $1^2\Pi_{x,y}$ at R = 20 a_0.

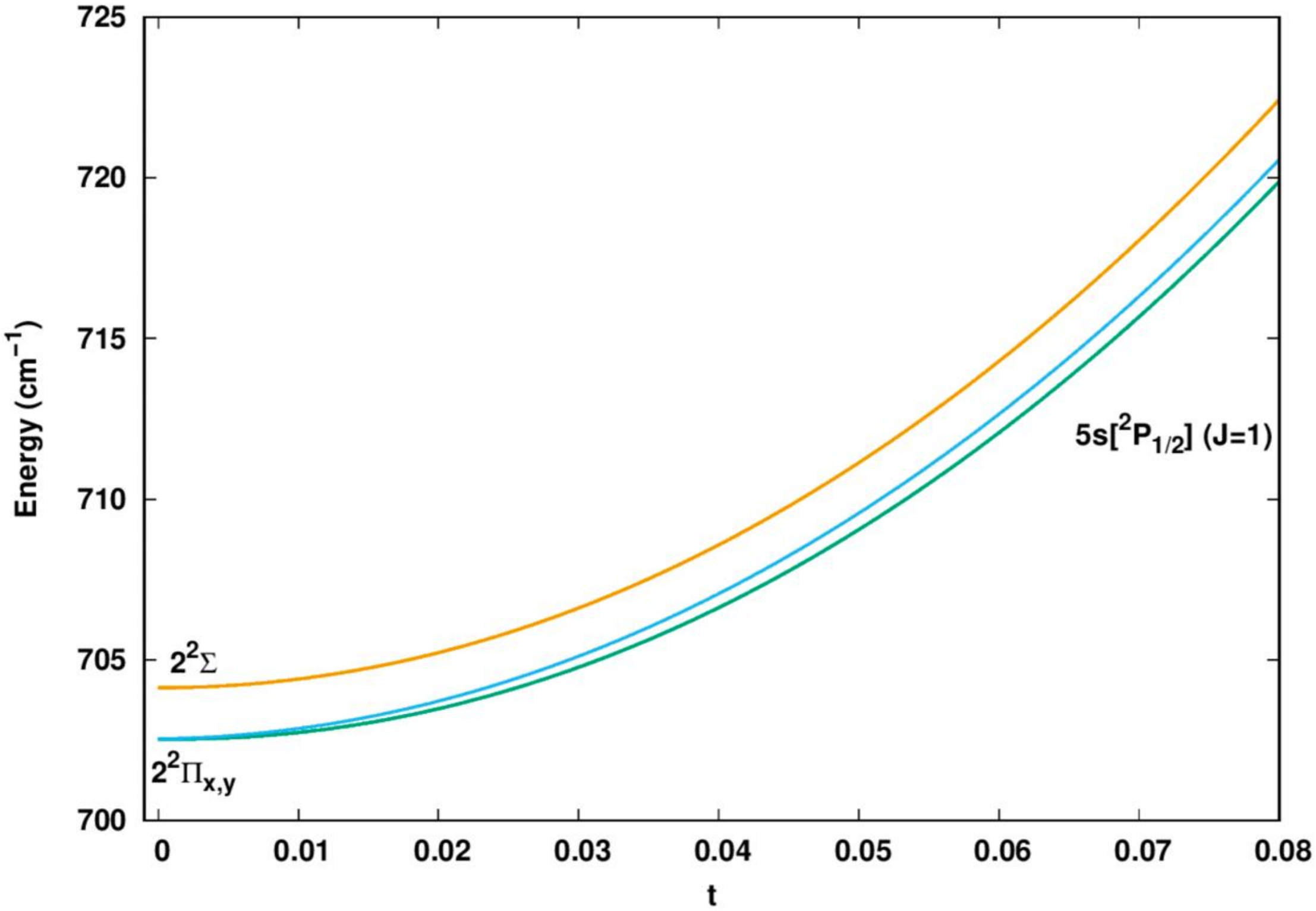

Fig S9. The spin-orbit splitting of $2^2\Sigma$ and $2^2\Pi_{x,y}$ at R = 20 a_0.

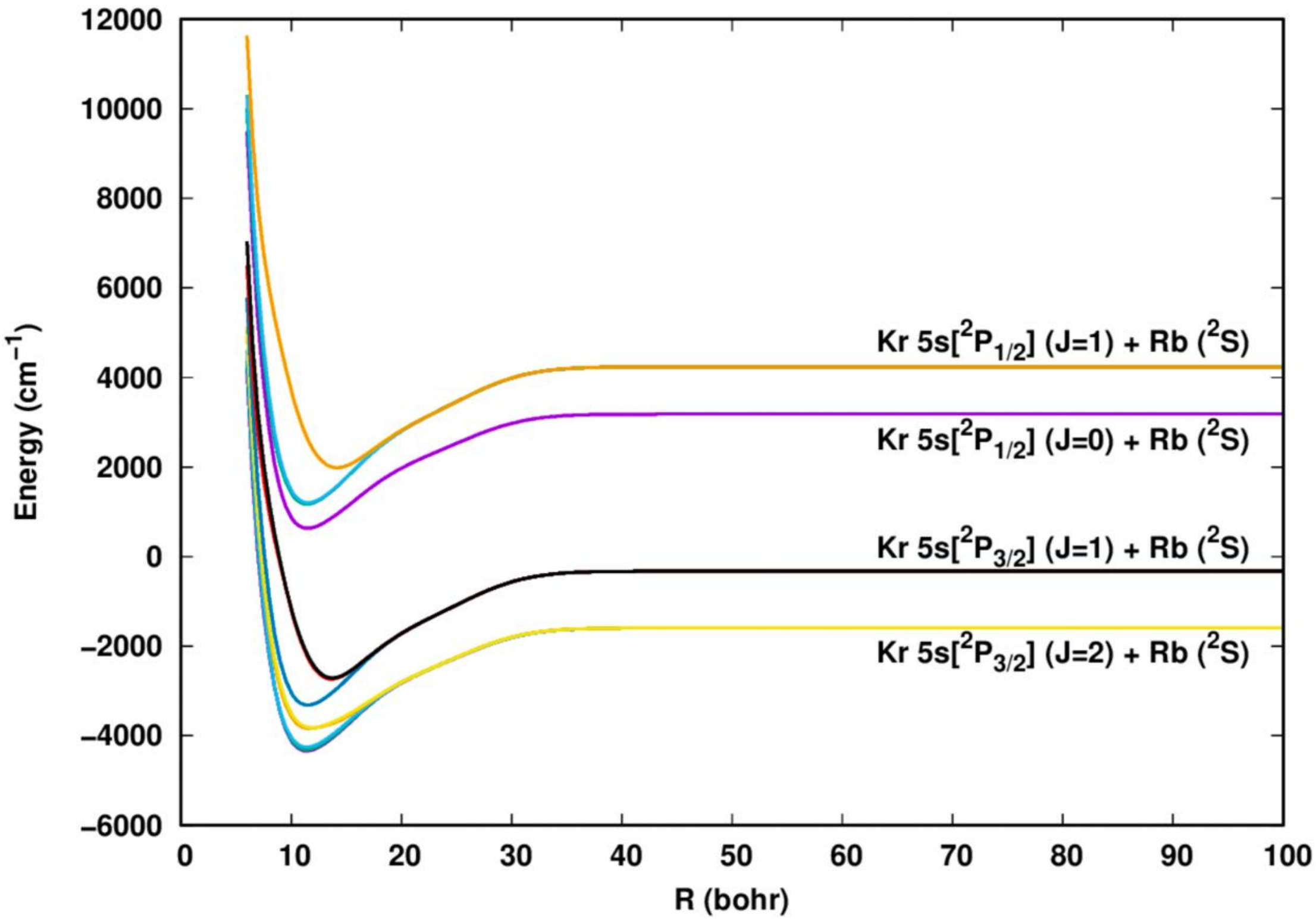

Fig S10. **Potential energy curves of Kr* + Rb with spin-orbit splitting.**

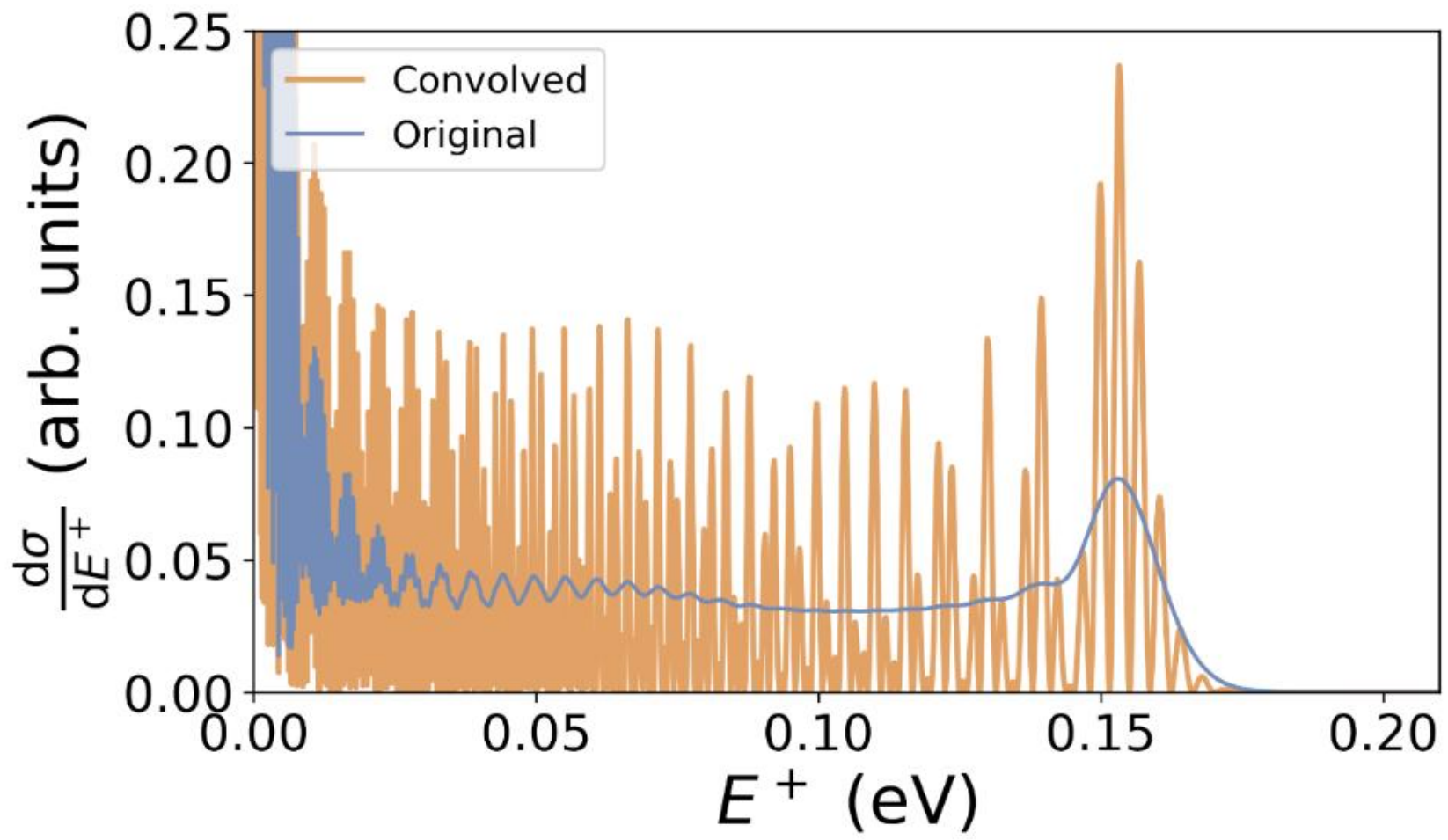

Fig S11. Theoretical ion energy distribution calculation incorporating energy broadening effects through Gaussian convolution.

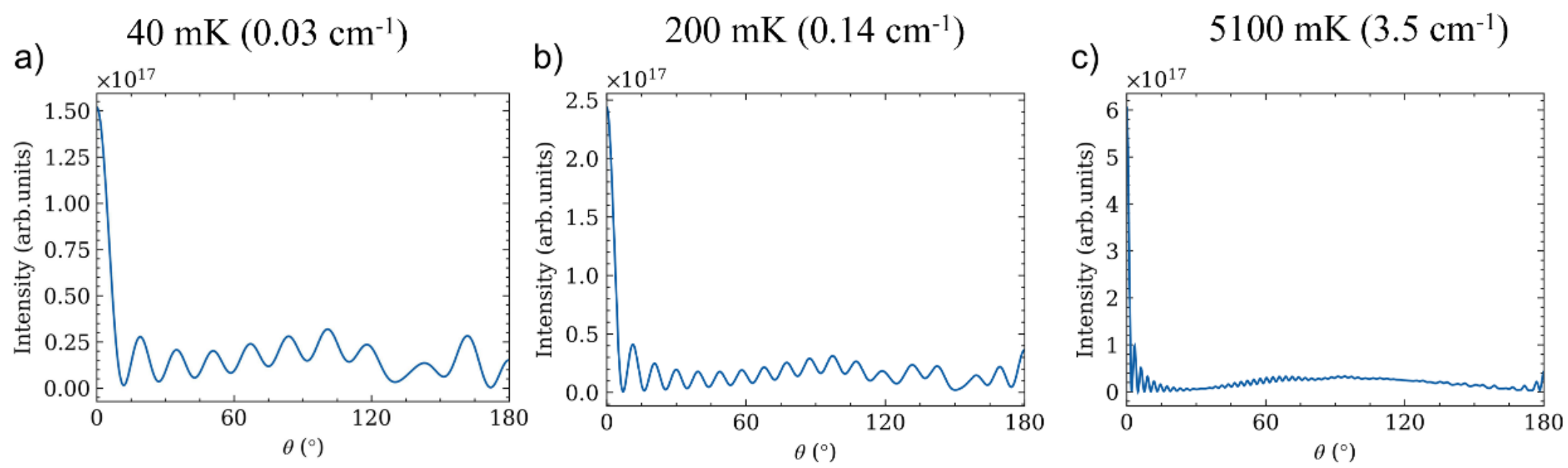

Fig S12. Angular DCS at different collision temperature. a) 40 mK, b) 200 mK, c) 5100 mK.

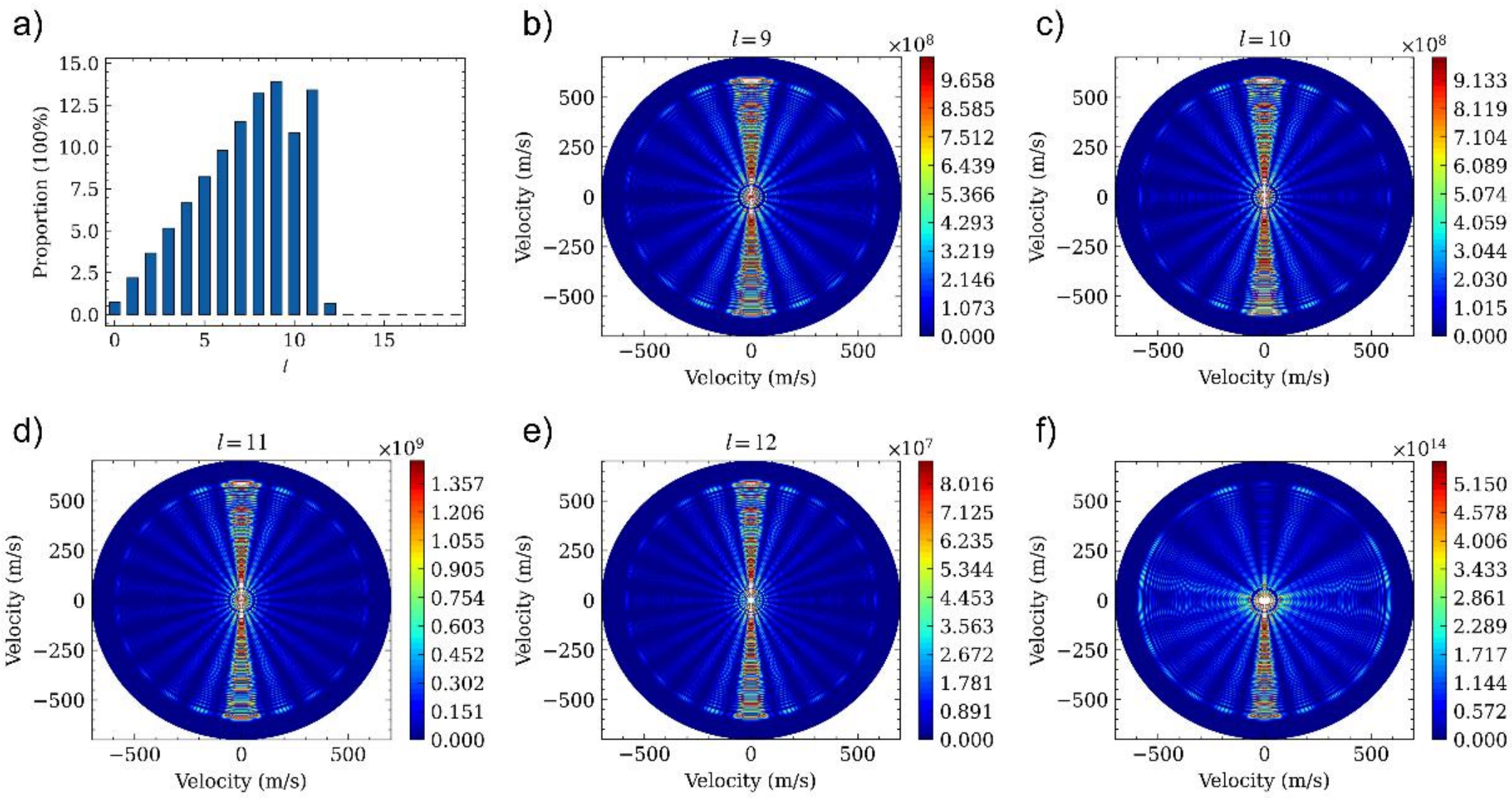

Fig. S13. Example of partial-wave DCS superposition. a) Relative contribution of single partial-wave DCS to the ICS (normalized to 100%). b-e) Angular-resolved ion velocity distributions for angular momentum quantum numbers $l = 9, 10, 11, 12$ respectively, with the vertical downward direction corresponding to the forward scattering direction. f) The total DCS image. The initial collision temperature is 40 mK.

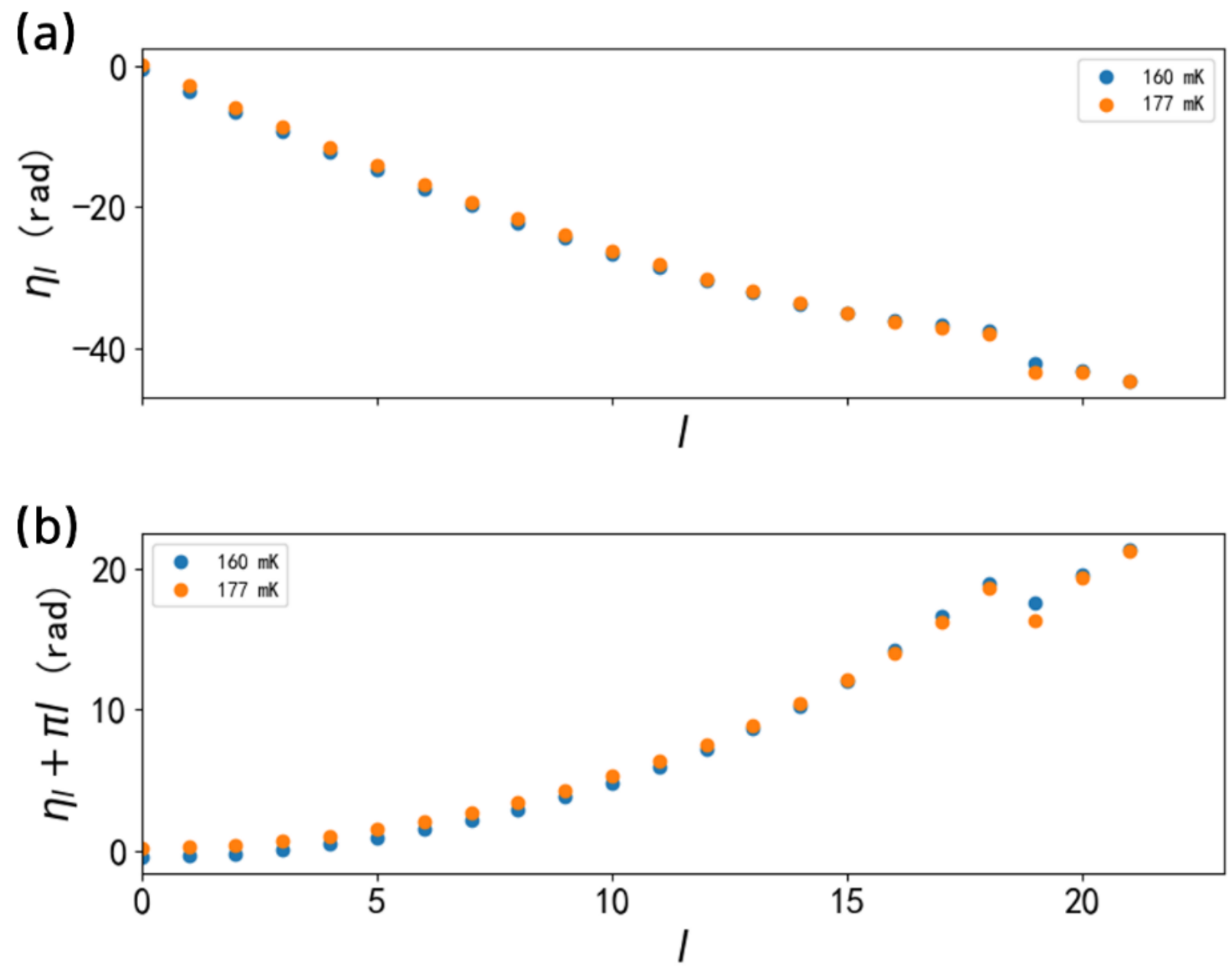

Fig S14. An example of phase shifts. The complex phase **(a)** η_l and **(b)** $\eta_l + \pi l$ of the S-matrix elements in different partial wave at 160 mK and 177 mK. The emitting energy of Rb^+ (E^+) is 0.12 eV.

Table S1. Key parameters of Kr^* + Rb MLR PEC

D_e (cm^{-1})	2743.84
r_e (Å)	6.01867
C_6 (cm^{-1}·$Å^6$)	2.841×10^6 (*ab initio*)<br>2.725×10^7 (exp fit)

Table S2. Parameters of Kr + Rb^+ PEC

$\alpha_{RgD}(\text{Å}^3)$	2.48[a]
$\alpha_{RgQ}(\text{Å}^5)$	3.97[a]
$\alpha_{RgO}(\text{Å}^7)$	16.35[a]
$C_6(e^2 a_0^5)$	83.31[b]
$C_8(e^2 a_0^7)$	1936[b]
$B_{Rg}(e^{-1}\text{Å}^6)$	-6.53[a]
$\gamma(e^{-2}\text{Å}^7)$	30.2[a]
Z	1.055[c]
$A(\text{cm}^{-1})$	1.078×10^{8}[c]
$b(\text{Å}^{-1})$	3.412[c]

a: see Ref (*44*), b: see Ref (*45*), c: see Ref (*46*).
